# A Bioinstructive Injectable Hydrogel for Enhancing Intrinsic Regeneration through Cell Recruitment and Training

**DOI:** 10.1002/advs.202514549

**Published:** 2025-12-23

**Authors:** Yurim Kim, Young‐Min Kim

**Affiliations:** ^1^ Center for Biomaterials Biomedical Research Institute Korea Institute of Science and Technology Seoul 02792 Republic of Korea; ^2^ Division of Bio‐Medical Science and Technology KIST School University of Science and Technology Seoul 02792 Republic of Korea

**Keywords:** in situ tissue regeneration, injectable hydrogel, intrinsic regeneration, stem cell recruitment, stem cell training

## Abstract

Tissue regeneration requires a precisely coordinated cascade of biological events—including stem cell homing, adhesion, proliferation, and differentiation—within a supportive and dynamic microenvironment. While numerous biomaterials have been designed to modulate individual regenerative processes, there is a need for a single, clinically viable platform that can synchronously modulate multiple regenerative events. Here, the study presents a strategically engineered injectable hydrogel that recapitulates this cascade by coordinating stem cell recruitment, matrix integration, and subsequent cellular development within a single localized system. The hydrogel is composed of amphiphilic, temperature‐responsive poly(organophosphazenes) (P) conjugated with polyethyleneimine (PP), enabling the co‐loading of laminin and stromal cell‐derived factor 1‐alpha (SDF‐1α) through ionic and hydrophobic interactions. The PP hydrogel exhibits thermosensitive sol–gel transition, sustained SDF‐1α release, and prolonged laminin retention. In vitro migration, adhesion, and proliferation assays confirm that the hydrogel enhanced stem cell recruitment and integration into the matrix. In a hindlimb ischemia mouse model, local hydrogel administration improves perfusion recovery and promotes robust angiogenesis. Together, these findings suggest that the hydrogel can coordinate several regenerative processes within a localized environment, supporting improved tissue repair in the studied model.

## Graphical abstract

A bioinstructive injectable hydrogel enables sustained release of stromal cell–derived factor‐1 alpha (SDF‐1α) and stable retention of laminin through ionic and hydrophobic interactions. This dual‐signaling matrix promotes endogenous stem cell recruitment and training, supports angiogenesis, and enhances intrinsic regenerative processes in ischemic tissue.

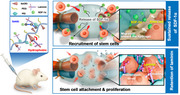

## 1 Introduction

Tissue regeneration is a complex and highly coordinated process involving stem cell homing, adhesion, proliferation, and differentiation.^[^ ^1^, ^2^ ^]^ Upon injury, various chemokines and growth factors are released, creating a chemotactic gradient that recruits endogenous stem or progenitor cells to the damaged site.^[^ ^3^, ^4^ ^]^ Once recruited, extracellular matrix (ECM) components engage integrins on the cell surface to strengthen cell adhesions and promote stable anchorage within the regenerative niche.^[^ ^5^, ^6^ ^]^ Following adhesion, the proliferative expansion of these cells replenishes the pool of cells at the injury site; then finely regulated differentiation of these cells into tissue‐specific lineages ultimately restores the structural and functional integrity of the tissue.^[^ ^7^, ^8^ ^]^ Failure in any of these stages—either insufficient recruitment, unstable adhesion, limited proliferation, or impaired differentiation—can lead to suboptimal regeneration or pathological outcomes such as fibrosis or scar formation.^[^ ^9^, ^10^ ^]^ Therefore, successful tissue regeneration requires an integrated microenvironment that supports each of these stages in a coordinated manner.

Extensive efforts have been made to enhance tissue regeneration by engineering biomaterials with integrated bioactive cues that target distinct phases of the regenerative cascade.^[^ ^11^, ^12^, ^13^, ^14^ ^]^ For instance, chemokine‐functionalized scaffolds have been designed to recruit endogenous stem cells, ECM‐derived motifs to facilitate adhesion, and growth factors to drive proliferation and lineage‐specific differentiation.^[^ ^15^, ^16^, ^17^ ^]^ Among these, SDF‐1α is widely recognized as a potent chemokine for mobilizing and homing stem/progenitor cells to ischemic or injured tissues,^[^ ^18^, ^19^ ^]^ while laminin, a key basement membrane protein, provides integrin‐binding motifs that reinforce adhesion and promote lineage‐specific differentiation.^[^ ^20^, ^21^ ^]^ These two factors represent complementary regulators of regenerative events and illustrate the potential of combinatorial strategies for recapitulating the regenerative cascade. However, most existing materials target individual stages of the regenerative pathway, and it remains challenging to develop strategies that can recapitulate the full complexity of the native microenvironment required to achieve complete and efficient tissue repair.^[^ ^22^ ^]^

To achieve such integration, numerous multi‐factor delivery systems—such as chemical conjugation, nanoparticle encapsulation, and layer‐by‐layer (LbL) assembly—have been explored to co‐deliver bioactive molecules with distinct functions.^[^ ^23^, ^24^, ^25^ ^]^ While these approaches partially replicate the complexity of native tissue repair, they often require labor‐intensive fabrication, risk altering molecular conformation, or lead to heterogeneous factor distribution that compromises bioactivity. Among the available material platforms, injectable hydrogels have emerged as a particularly promising alternative, offering structural tunability, minimally invasive delivery, and localized, sustained release of multiple bioactive cues.^[^ ^26^, ^27^, ^28^ ^]^ Furthermore, if designed to promote both stem cell homing and stable adhesion, such hydrogels could serve as an ideal microenvironmental scaffold that coordinates the multistage processes of intrinsic tissue repair.

**Figure 1:**
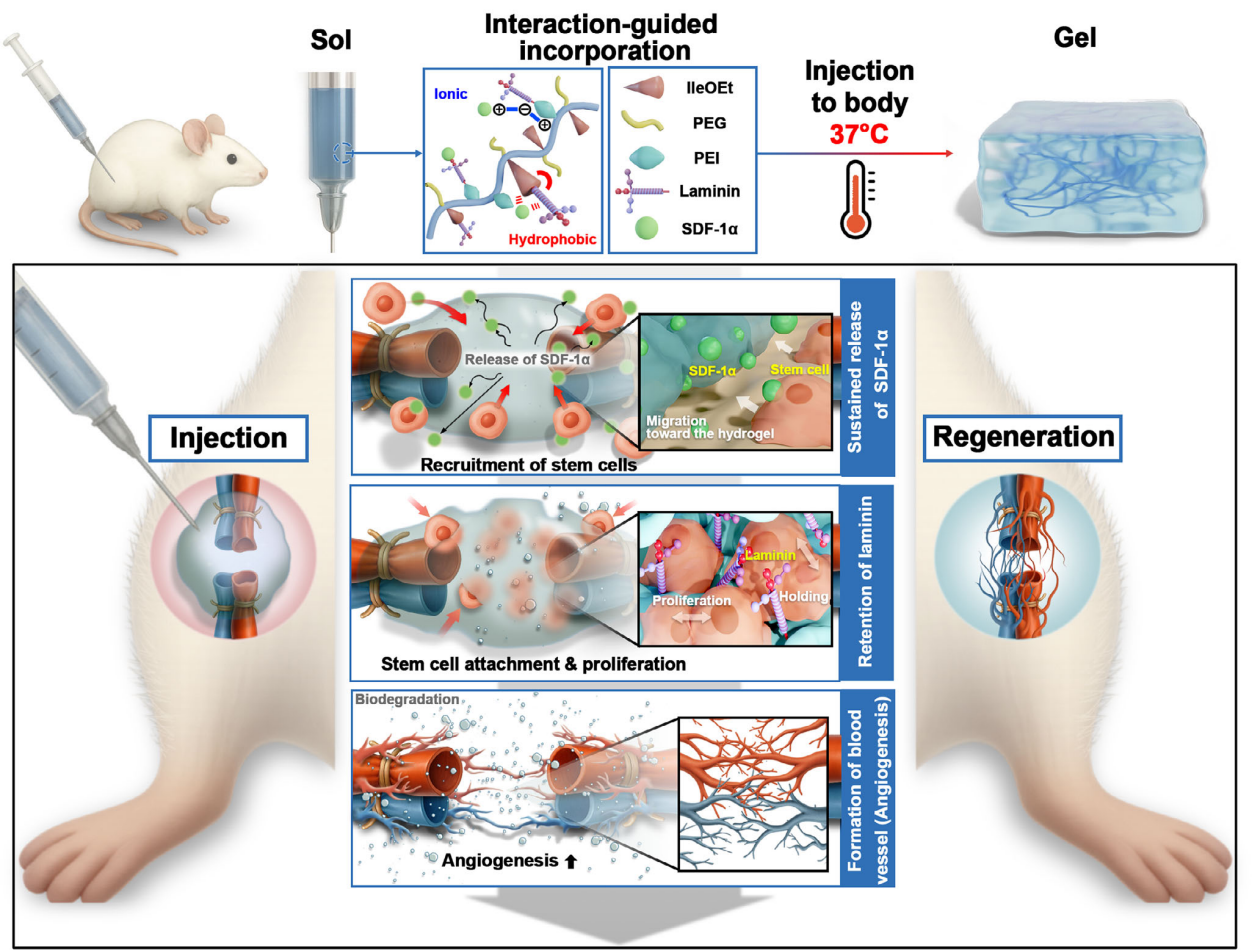
Schematic diagram of the injectable hydrogel system that promotes cell migration and guiding. This system was designed via ionic and hydrophobic interactions to mimic the body's natural regenerative system. The hydrogel recruits endogenous stem cells via SDF‐1α and promotes their proliferation through laminin, effectively facilitating angiogenesis in hindlimb ischemic tissue.

Here, we developed an injectable intrinsic regeneration‐mimicking system designed to recruit and train stem cells, enabling highly effective restoration (**Figure** **1**). This system leverages a novel hydrogel complex composed of amphiphilic, temperature‐responsive PP, which introduces a positive charge to enable the co‐loading of negatively charged laminin and positively charged SDF‐1α through ionic and hydrophobic interactions. Upon minimally invasive injection, the hydrogel facilitates the sustained release of SDF‐1α to initiate stem cell recruitment, whereas laminin exhibits prolonged retention within the matrix owing to its larger molecular size and stronger multivalent ionic and hydrophobic interactions with the PP network. These differential release kinetics provide a stable adhesive interface that promotes robust cellular attachment and facilitates lineage‐specific differentiation. In a hindlimb ischemia model, the hydrogel demonstrated potent vascular regeneration, and its ability to modulate multiple regenerative cues suggests potential utility for supporting tissue repair.

## 2 Results and Discussion

### 2.1 Synthesis and Characterization of PP

The thermosensitive P hydrogel was designed to be injected in liquid form, allowing conformal integration into irregular 3D tissue defects with minimal invasiveness.^[^ ^29^, ^30^ ^]^ To enable the ionic interaction loading of anionic extracellular matrix components such as laminin, **P**—composed of hydrophobic L‐isoleucine ethyl ester (IleOEt), hydrophilic α‐amino‐ω‐methoxy poly(ethylene glycol) (AMPEG), and carboxylic acid groups—was further modified by conjugating **p**olyethyleneimine (PEI) through an amide‐coupling reaction. The structure of PP was confirmed via ^1^H‐NMR, where a peak at 2.7 ppm indicated the successful conjugation of PEI to the carboxylic acid terminus of P (Figure S1, Supporting Information). In the ^13^C‐NMR spectrum of P, a signal at 30.94 ppm corresponding to the α‐methylene group adjacent to free –COOH was observed (Figure S2, Supporting Information). This peak disappeared in PP due to conversion of –COOH into amide linkages. Fourier Transform Infrared Spectroscopy (FT‐IR) spectra further verified this conjugation, showing a decrease of the –COOH band (≈1720 cm^−1^) accompanied by the emergence of amide I (≈1650 cm^−1^) and amide II (≈1550 cm^−1^) bands (Figure S3, Supporting Information). Gel Permeation Chromatography (GPC) analysis in THF using polystyrene standards revealed a shift in molecular‐weight distribution from P (Mn ≈ 1867, Mw ≈ 2191, PDI 1.17) to PP (Mn ≈ 1289, Mw ≈ 1356, PDI 1.05), supporting successful PEI conjugation despite potential solvent–polymer interaction effects (Figure S4, Supporting Information). The conjugation ratio of PEI was estimated using the ninhydrin assay (Figure S5, Supporting Information), which revealed that the side chains, comprising IleOEt, AMPEG, carboxyl acid, and PEI, were distributed in proportions of 71.5%, 16.5%, 8.5%, and 3.5%, respectively.

Cryogenic scanning electron microscopy (Cryo‐SEM) analysis revealed that PP hydrogel possessed an interconnected porous microstructure with an average pore size of 5.40 ± 0.78 µm (Figure S6, Supporting Information), a morphology considered favorable for mesenchymal stem cell (MSC) infiltration and subsequent tissue ingrowth. Given the necessity for cytocompatibility in regenerative scaffolds, thiazolyl blue tetrazolium bromide (MTT) assays confirmed nearly 100% cell viability for both fibroblasts and MSCs at polymer concentrations up to 1000 µg mL^−1^ (Figures S7 and S8, Supporting Information). Furthermore, in line with our previous findings, the P hydrogel degraded at ≈2% per day, indicating that the polymer concentration remained well above the degraded fraction throughout the culture period.^[^ ^31^ ^]^ Collectively, these results demonstrate that the PP hydrogel combines a cell‐permissive architecture with excellent cytocompatibility, supporting its suitability as a safe and effective scaffold for regenerative applications.

### 2.2 Functionalization of PP to Facilitate Stem Cell Homing and Attachment: Ionic and Hydrophobic Interaction and Temperature‐Responsive Gelation

**Figure 2:**
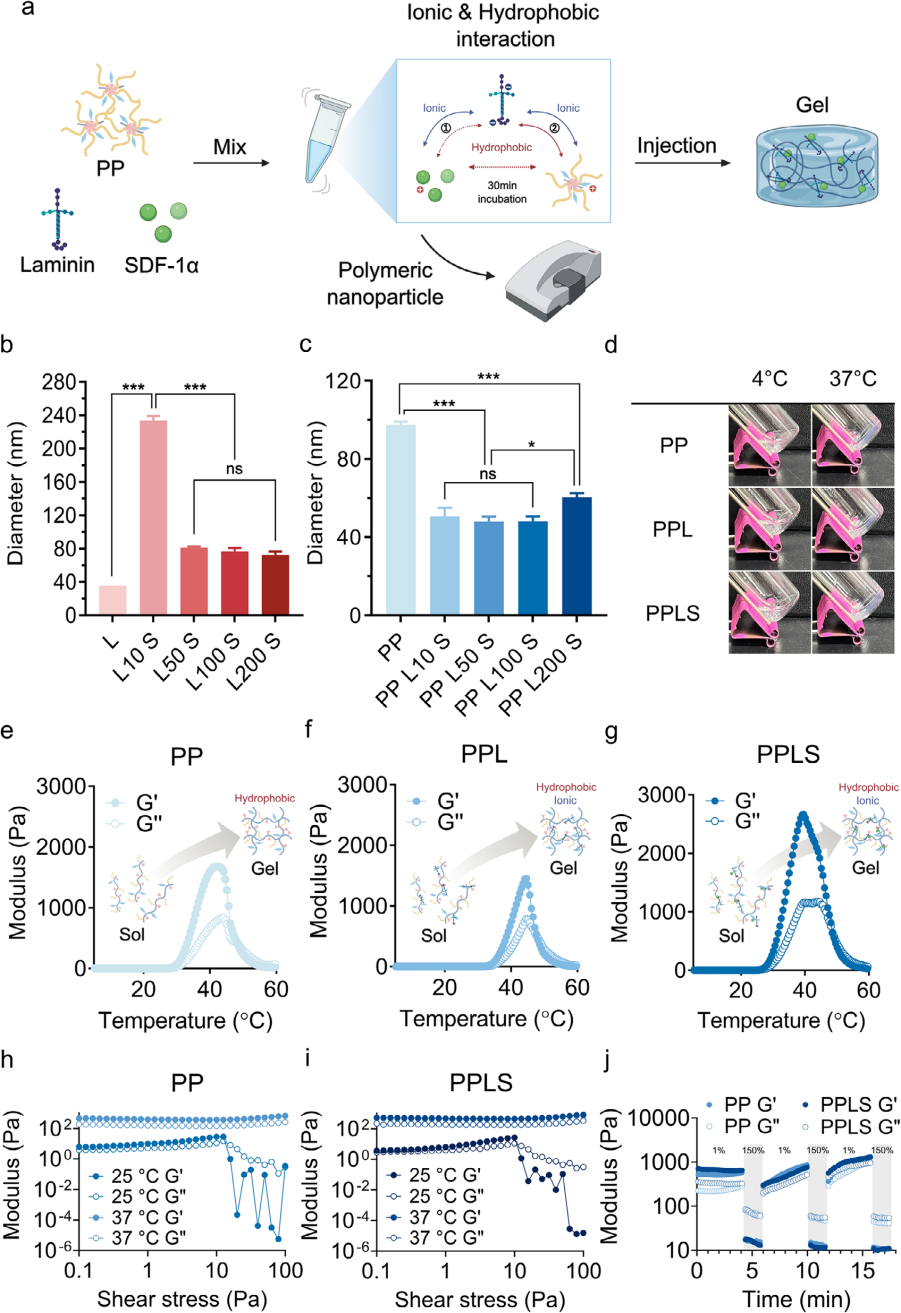
Preparation and characterization of the PPLS hydrogel. a) Illustration of the procedure for producing the PPLS hydrogel system. b) Size of the LS complexes as a function of the laminin concentration (n = 3). c) Size of the PPLS complexes as a function of the laminin concentration (n = 3). d) Images of the sol‐gel transition of the PP, PPL, and PPLS hydrogels at 4 and 37 °C. e–g) Temperature‐dependent rheological data of the 10 wt.% PP, PPL, and PPLS hydrogels. h,i) Shear‐thinning behavior of PP and PPLS by shear stress sweep at 25 and 37 °C. j) Self‐healing behavior of PP and PPLS under alternating low (1%) and high (150%) shear strain at 37 °C. *p < 0.01 and ***p < 0.0001.

We developed a hydrogel system that emulates the body's natural regeneration mechanism by simply mixing negatively charged laminin with positively charged SDF‐1α, followed by the addition of a positively charged nontoxic polymer backbone to enhance stem cell recruitment and retention (**Figure** **2**a). This system also engages in hydrophobic interactions with specific domains of the incorporated molecules, further stabilizing the complex, with differential ionic and hydrophobic affinities contributing to the selective incorporation and distinct release kinetics of laminin and SDF‐1α. The polymer exhibited amphiphilic properties, which enabled it to self‐assemble and form polymeric micelle structures.^[^ ^32^ ^]^ In previous studies, the size of polymeric nanoparticles decreased after growth factors were loaded owing to ionic and hydrophobic interactions.^[^ ^29^, ^33^ ^]^ Similarly, we expected that the ionic and hydrophobic interactions between PP, laminin, and SDF‐1α would affect nanoparticle size, attributed to their respective charges. Interactions within the PP, laminin, and SDF‐1α complex (PPLS) were confirmed by observing the changes in size after mixing varying concentrations of laminin and SDF‐1α (Figure 2b,c). The SDF‐1α concentration was fixed at 200 ng mL^−1^, a range previously shown to promote sustained stem cell homing for up to three weeks,^[^ ^34^, ^35^ ^]^ enabling reliable evaluation of laminin‐dependent complex formation. As the concentration of laminin increased, the size of the laminin and SDF‐1α complex (LS) gradually decreased from 233.5 to 72.5 nm. The complex condensed at higher concentrations; however, no significant difference in the particle size was observed from laminin 50 µg mL^−1^ (L50) to 200 µg mL^−1^ (L200). When LS was mixed with PP, and the laminin concentration increased to laminin 100 µg mL^−1^ (L100), the size of the complex decreased from 97.5 to 48.0 nm. However, the size increased to 60.3 nm at L200, indicating that concentrations higher than L100 could not form more stable complexes than L100 could. In the LS complexes, the ζ‐potential decreased progressively as the laminin concentration increased, reflecting the increasing contribution of the anionic laminin (Figure S9a, Supporting Information). In the PPLS complexes, the initial mixing with cationic PP largely neutralized the surface charge, and although the ζ‐potential showed a slight downward trend with increasing laminin concentration, the differences among the higher laminin groups (L50–L200) were not statistically significant (Figure S9b, Supporting Information). Transmission electron microscopy (TEM) further confirmed that the morphology and particle size of the complexes were consistent with the DLS measurements (Figure S10, Supporting Information). These results showed that laminin and SDF‐1α can interact with PP through ionic and hydrophobic interactions, and that varying laminin concentrations affect the strength of such interactions, with L100 forming the most condensed complex.

The temperature‐dependent gelation of PPLS was verified through rheological analysis to assess its feasibility as an injectable hydrogel system. The polymer solution transitioned from the liquid state at 4 °C to a gel structure at 37 °C, and this gelation persisted when laminin and SDF‐1α were mixed into the PP (Figure 2d). Furthermore, the storage and loss moduli of PP, PP and laminin complex (PPL), and PPLS were found to validate temperature‐responsive gelation at temperatures ranging from 4 to 60 °C (Figure 2e–g). PP, PPL, and PPLS exhibited sol‐gel transitions within a specific temperature range (26–32 °C) and retained their gel state at body temperature. The shear‐thinning behavior, a key property for injectability, was evaluated under varying shear stress at 25 and 37 °C (Figure 2h,i).^[^ ^36^ ^]^ Both PP and PPLS hydrogels exhibited a marked decrease in modulus with increasing shear stress at 25 °C, consistent with typical shear‐thinning behavior. At 37 °C, however, both hydrogels retained their gel‐like states up to 100 Pa, suggesting enhanced mechanical stability under physiological conditions. Injectability of the hydrogel was further confirmed both in vitro and in vivo: in vitro, 4 mg mL^−1^ of nile red was added to enhance visualization during injection into 37 °C water, and in vivo, a single subcutaneous injection into the mouse hindlimb was performed using a 31‐G insulin syringe (Movies S1 and S2, Supporting Information). In both settings, the hydrogel was smoothly extruded through the fine needle in its sol state and rapidly gelled at 37 °C, maintaining its structural integrity at the target site. To further investigate the mechanical resilience of the hydrogels, a self‐healing test was conducted by alternating low (1%) and high (150%) strain cycles (Figure 2j). Both PP and PPLS hydrogels exhibited rapid and repeatable recovery of G′ following high‐strain disruption, indicative of intrinsic self‐healing capacity. Notably, PPLS showed a slightly higher initial G′ prior to deformation, reflecting a more reinforced network. However, its recovery extent was marginally reduced compared to PP, likely due to the increased structural complexity arising from the incorporation of biofunctional cues such as laminin and SDF‐1α.^[^ ^37^, ^38^ ^]^ These moieties participate in reversible non‐covalent interactions, including ionic and hydrophobic bonding, which contribute to mechanical resilience but may limit complete network reformation upon repeated strain.^[^ ^39^ ^]^ Despite this, PPLS maintained sufficient structural integrity under dynamic conditions, supporting its potential to facilitate cellular infiltration, adhesion, and differentiation post‐injection. Cryo‐SEM analysis further revealed that PP and SDF‐1α complex (PPS), PPL, and PPLS hydrogels possessed interconnected porous microstructures with average pore sizes of approximately 4–6 µm (PPS: 5.24 ± 0.78 µm; PPL: 5.25 ± 0.56 µm; PPLS: 5.04 ± 0.75 µm), a morphology favorable for mesenchymal stem‐cell infiltration and subsequent tissue ingrowth (Figure S11, Supporting Information). Thus, these systems enable minimally invasive injection into target sites while maintaining their 3D structure and encapsulating laminin and SDF‐1α to support stem cell homing and guidance.

### 2.3 Comprehensive In Vitro and In Vivo Investigations of Laminin Retention and SDF‐1α Release from PPLS for Stem Cell Recruitment and Proliferation

**Figure 3:**
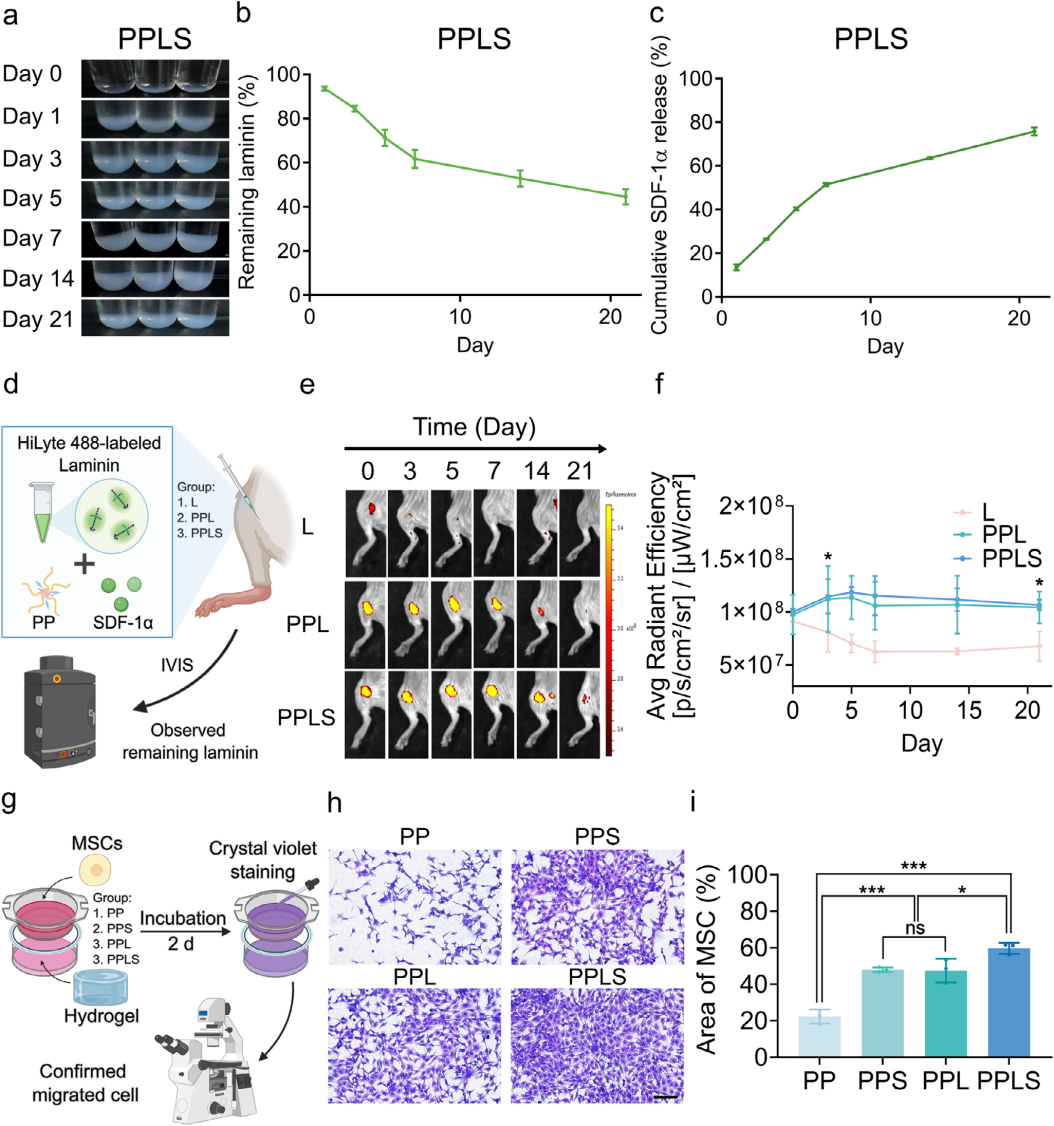
Assessment of stem cell homing facilitated by the sustained release of SDF‐1α and holding supported by the retention of laminin within the hydrogel. a) In vitro dissolution test for the PPLS hydrogel to evaluate laminin retention and sustained SDF‐1α release. b) In vitro laminin holding profile from the PPLS hydrogel (n = 3). c) In vitro profile of SDF‐1α release from the PPLS hydrogel (n = 3). d) Schematic illustration of IVIS‐based in vivo tracking of laminin retention. e) Evaluation of laminin retention through IVIS imaging and green fluorescent‐tagged laminin in the L, PPL, and PPLS groups, in a hindlimb ischemic mouse model at 0, 3, 5, 7, 14, and 21 d post‐injection. f) Quantification of laminin retention (n = 3). Scale bar = 1000 µm. g) Schematic of MSC migration assay through PP, PPS, PPL, and PPLS hydrogel. h) Microscopy images of MSC migration after 48 h. Scale bar = 500 µm. i) Quantification of the area of migrating cells (n = 3). *p < 0.01 and ***p < 0.0001.

To mimic natural regeneration cues by facilitating stem cell recruitment and proliferation at the injury site, the PPLS should be injected at a concentration that forms the most stable complex, which ensures the continuous release of SDF‐1α and retention of laminin within the hydrogel for an optimized period.^[^ ^40^, ^41^, ^42^ ^]^ We assessed the post‐injection maintenance of the PPLS hydrogel within the body, via mass loss tests to indirectly assess its stability, and also assessed the release of laminin and SDF‐1α to determine their long‐term retention within the hydrogel (**Figure** **3**). Laminin was expected to exhibit more sustained release than SDF‐1α, owing to its larger polymeric structure and multivalent interactions with the PP network, including partial affinity for the hydrophobic domains of the PP.^[^ ^20^, ^43^, ^44^ ^]^ The PPLS hydrogel remained structurally stable for up to 21 d in vitro (Figure 3a). Consistently, in vivo subcutaneous injection into the hindlimb revealed that the hydrogel maintained its shape and appeared tissue‐integrated at 21 d, while by 30 d, it was completely degraded and seamlessly merged with host tissue without any apparent inflammatory response, as confirmed by H&E staining (Figure S12, Supporting Information). These findings indicate that PPLS hydrogel gradually undergoes controlled degradation in vivo while maintaining sufficient integrity to support early regenerative events. As expected, laminin was released more slowly than was SDF‐1α, with ≈40% retained in the hydrogel, whereas nearly 80% of SDF‐1α was released over the same period (Figure 3b,c). Previous reports have indicated that 10–100 µg mL^−1^ laminin has regenerative effects when retained within the scaffold for approximately 2 weeks, whereas 100–200 ng mL^−1^ SDF‐1α is commonly used to promote stem cell homing through gradual release over a three‐week period.^[^ ^34^, ^35^ ^]^ Therefore, the continuous release of 200 ng mL^−1^ SDF‐1α over a prolonged period facilitates the steady recruitment of the surrounding stem cells, while ≈40 µg mL^−1^ laminin retained within the hydrogel promotes their proliferation. In addition, in vivo IVIS imaging of HiLyte‐labeled laminin revealed rapid signal loss when administered alone, whereas laminin delivered within the PPL or PPLS hydrogel exhibited prolonged retention for up to 21 days (Figure 3d–f; Figure S13, Supporting Information). Given that cells typically require 2–3 weeks to attach completely and differentiate, these results underscore the importance of extended laminin persistence in potentially promoting tissue regeneration.^[^ ^40^ ^]^ Consequently, the PPLS hydrogel enables the continuous release of 80% of SDF‐1α and retains 40% of laminin in vitro while maintaining intact laminin in vivo for 21 d, facilitating stem cell recruitment and differentiation within the hydrogel in a manner that resembles the body's intrinsic regenerative system.

To ensure the continuous recruitment of stem cells by SDF‐1α, requires its stability and efficacy within the hydrogel, and its released form must also maintain its efficacy. This effect was evaluated through MSC migration (Figure 3g,h; Figure S14, Supporting Information) and scratch assays using human umbilical vein endothelial cells (HUVECs) (Figure S15, Supporting Information). In the MSC migration assay, the PPLS hydrogel was positioned in the lower chamber with complete medium, while the MSCs were seeded in the upper chamber and incubated for 48 h. Compared with other groups, the PP group presented a lower migration rate, whereas the PPLS group exhibited the highest migration rate of 60% (Figure 3i). The PPL and PPS groups exhibited a similar trend, which can be attributed to the favorable microenvironment established by ECM presentation, providing adhesive cues and supporting cell–matrix interactions that promote motility.^[^ ^45^, ^46^ ^]^ To further verify that SDF‐1α–mediated migration occurs via the CXCR4 pathway, we treated the PPLS group with the CXCR4 antagonist AMD3100, which markedly suppressed MSC infiltration. Similarly, in the scratch assay, the PPLS group showed the most significant wound healing effect after 24 h. These results indicate that SDF‐1α can induce MSC migration, with the combined system proving to be more effective than the other systems alone.

**Figure 4:**
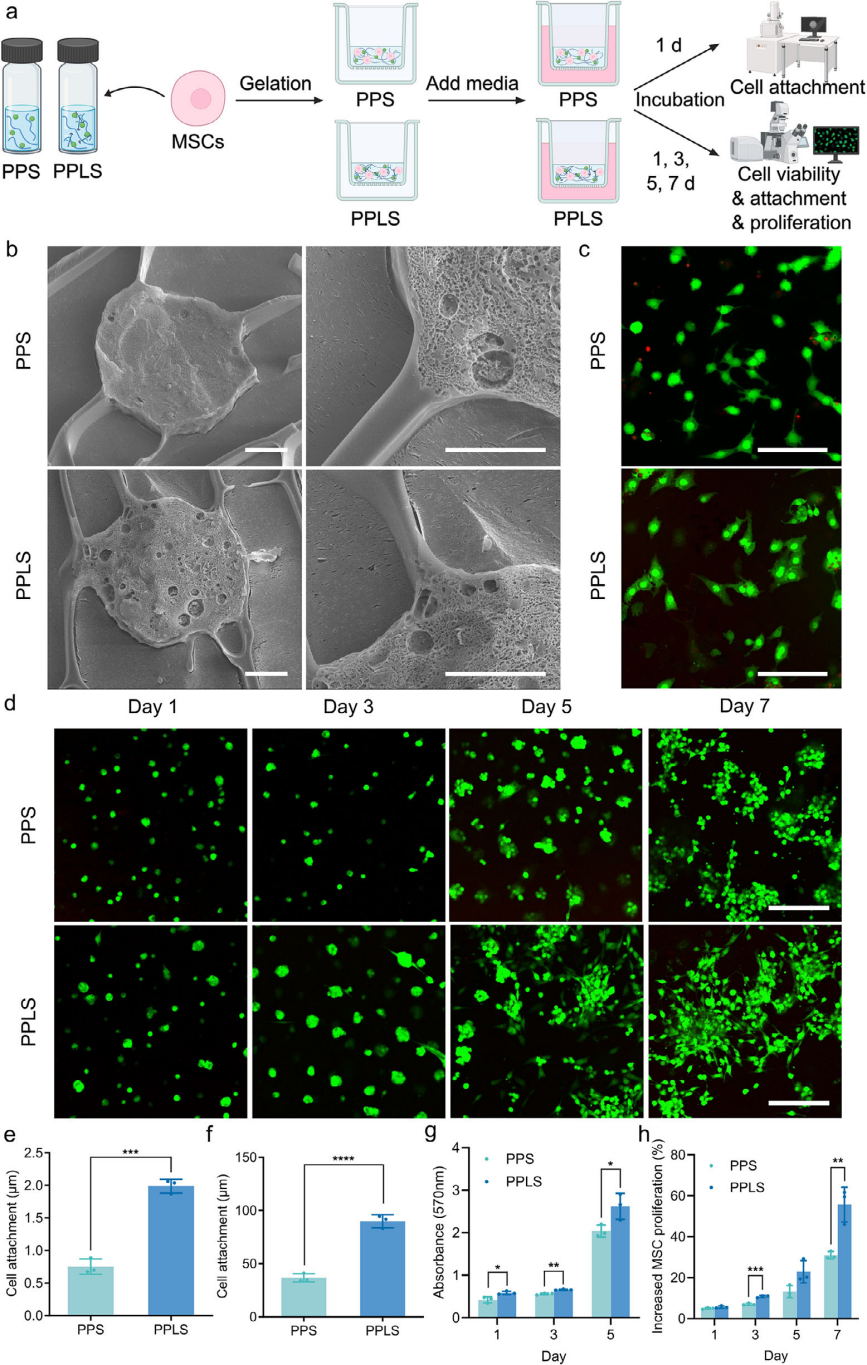
Analysis of the effects of laminin within the PPLS hydrogel on stem cell adhesion and proliferation. a) Schematic illustration of the cryo‐SEM and live/dead assay. b) Evaluation of MSC attachment within the PPS and PPLS hydrogels using cryo‐SEM imaging after 1 d of incubation. Scale bar = 5 µm. c) Evaluation of MSC attachment within the PPS and PPLS hydrogels using live/dead staining after 3 d of incubation. Scale bar = 200 µm. d) Confocal images for assessing the effect of laminin on cell proliferation. Scale bar = 200 µm. e,f) Quantification of cell attachment using cryo‐SEM imaging and live/dead assays (n = 3). g,h) Quantification of cell proliferation using MTT and live/dead assays (n = 3). *p < 0.05, ***p < 0.0005, and ****p < 0.0001.

In addition, SEM and live/dead images were obtained to evaluate the cell adhesion facilitated by laminin within the hydrogel (**Figure** **4**). The hydrogels were mixed with the MSCs and incubated for 30 min to allow gelation, followed by further incubation periods of 1, 3, 5, and 7 d (Figure 4a). Compared with those in the PPS group, the MSCs in the PPLS group showed significantly greater adherence to the hydrogel by approximately 2.8‐fold (Figure 4b,e). A live/dead assay was also used to assess cell viability (Figure S16, Supporting Information) and attachment (Figure 4c,f) to the PPS and PPLS hydrogels after 3 d of incubation. Both groups demonstrated good biocompatibility with MSCs; however, the PPLS group exhibited greater cell viability owing to the presence of laminin. Notably, the cells embedded in the PPLS hydrogel had a stretched and elongated morphology, in contrast to the more spherical shape of the cells within the PPS hydrogel. Moreover, we evaluated MSC proliferation using MTT and live/dead assays over a 7 d incubation period (Figure 4g,h). The MSCs proliferated more rapidly and exhibited an extended, spread morphology in the PPLS group. These results indicate that the PPLS hydrogel mimics the body's natural regeneration system by enabling the sustained release and retention of SDF‐1α and laminin, which also demonstrates its potential for promoting the recruitment and proliferation of endogenous stem cells at the injury site to facilitate recovery.

### 2.4 Vascular Regeneration Effects in Hindlimb Ischemia Using the PPLS Hydrogel

**Figure 5:**
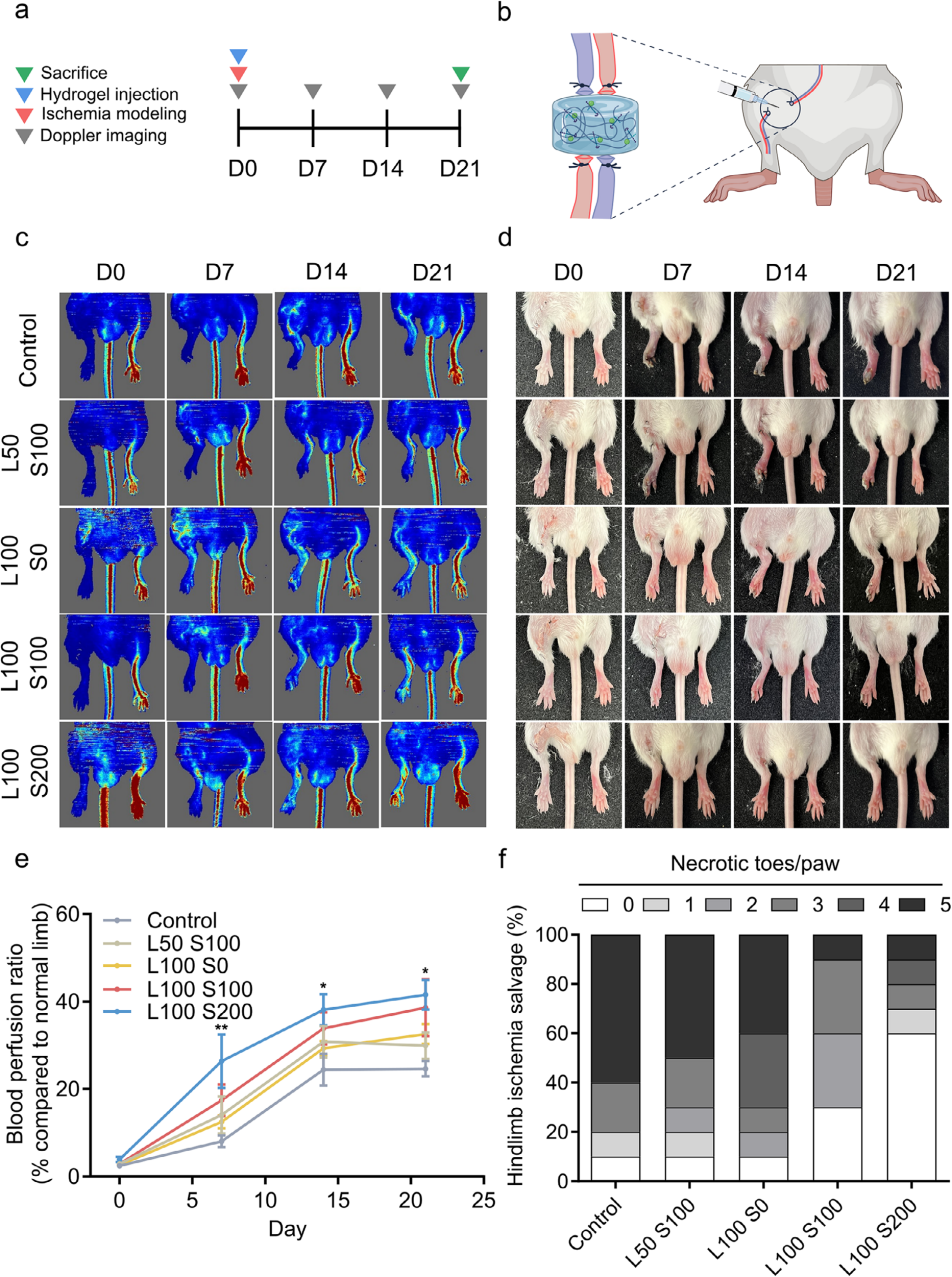
Regenerative evaluation in a mouse model of hindlimb ischemia. a) Schematic of the schedule for hindlimb modeling. b) Schematic diagram of the establishment and treatment of the hind limb ischemia model. c) Laser Doppler perfusion imaging (LDPI) of the hindlimb ischemia model. d) Representative images of mouse hindlimbs on the designated days. e) Blood perfusion ratios at 0, 7, 14, and 21 d post‐injection (n = 10). f) Fractional ratio of necrotic toes/paws in ischemic hind limbs 21 d post‐surgery (n = 10). *p < 0.01 and **p < 0.001.

The hindlimb ischemia model was used for in vivo experiments since the hydrogel system emulates the body's natural regenerative pathways to promote vascular regeneration by releasing SDF‐1α to recruit cells and utilizing laminin to facilitate the attachment and proliferation of recruited cells.^[^ ^19^, ^34^ ^]^ We established an ischemia mouse model to evaluate the therapeutic potential of PPLS hydrogels with varying concentrations of laminin and SDF‐1α in vivo. A preliminary test across multiple concentrations was performed to identify the most effective groups (Figure S17, Supporting Information), which were then expanded for the main study. The overall experimental timeline and injection procedure are illustrated in **Figure** **5**a,b. Mice received a single direct injection of the hydrogel into the ischemic site, and blood flow was monitored weekly by laser Doppler imaging until sacrifice at 21 d (Figure 5c–f). Laser Doppler imaging revealed no blood supply to the right limb in any of the groups on 0 d, indicating successful ischemic modeling (Figure 5c,e; Figure S18, Supporting Information). On 7 d, the blood flow in the limbs in the L100 S200 group recovered faster than that in the other groups did, and this trend was maintained for the entire 3‐week duration. Similar to the analysis of perfusion ratio, right hindlimb salvage was assessed on 21 d to evaluate regeneration. Nearly all the mice in the control group experienced complete toe and paw loss due to necrosis, whereas most of the mice in the L100 S200 group showed significant recovery compared with those in the other groups (Figure 5d,f). Typically, injections of a regenerative hydrogel system at two to four sites in the hindlimb exhibit angiogenic effects; however, our hydrogel system achieved comparable outcomes with only a single injection.^[^ ^47^, ^48^ ^]^ Given that L100 S200 effectively interacts with PP, it may facilitate superior regenerative effects. As a result, varying concentrations of laminin and SDF‐1α affect hydrogel interactions, thereby influencing hindlimb regeneration; the L100 S200 group demonstrated greater regenerative effects than the other groups did.

### 2.5 Analysis of Regeneration Mechanisms in a Hindlimb Ischemia Model

**Figure 6:**
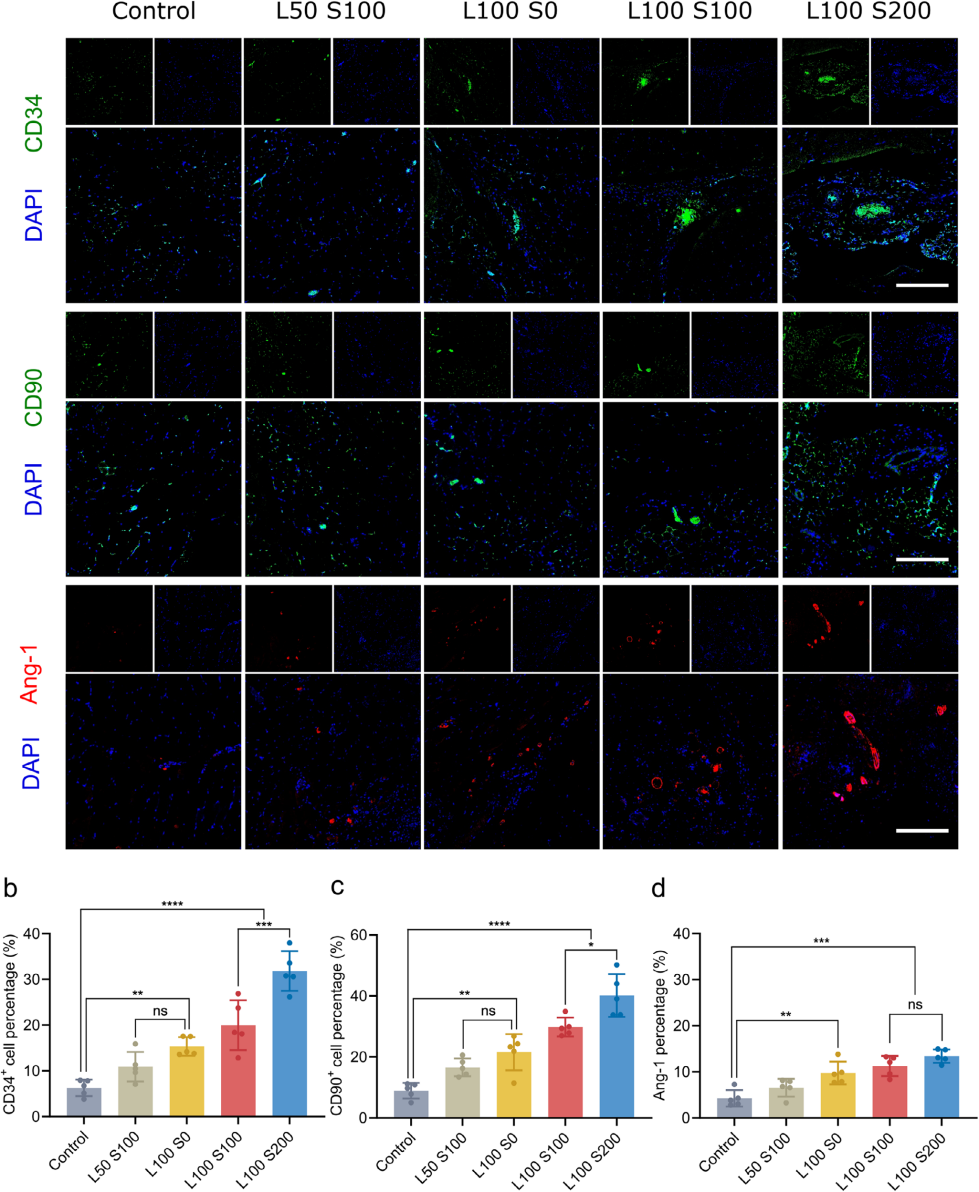
Recruitment and differentiation of endogenous stem cells. a) Immunofluorescence staining images of CD34, CD90, and Ang‐1. Scale bar = 200 µm. b) Quantitative analysis of the percentages of CD90^+^ cells (n = 5). c) Quantitative analysis of the percentages of Ang‐1 stained area (n = 5). d) Quantitative analysis of the Ang‐1‐stained area (n = 5). *p < 0.01, **p < 0.001, ***p < 0.0005, and ****p < 0.0001.

To determine whether the regeneration process was facilitated by the recruitment of endogenous stem cells, we performed staining for CD34 and CD90, which are well‐known MSC markers, to trace endogenous stem cells (**Figure** **6**a–c).^[^ ^49^ ^]^ All the groups contained CD34^+^ and CD90^+^ cells, with significantly greater numbers detected in the hydrogel group than in the control group. Consistent with the results of the ischemia test, L100 S200 had a higher number of CD34^+^ and CD90+ cells, suggesting that increased SDF‐1α and laminin concentrations could enhance the long‐term recruitment and retention of endogenous stem cells at the injury site. Additionally, we assessed the differentiation of the recruited stem cells into blood vessels using angiopoietin‐1 (Ang‐1) immunostaining (Figure 6a,d). In the control group, the presence of blood vessels was minimal. However, as the concentration of laminin increased, the expression level of Ang‐1 also increased, with the highest percentage observed in the L100 S200 group. This can be attributed to the interaction between laminin and cells, which induces the differentiation of recruited stem cells into blood vessels. The above results indicate that the hydrogel system composed of laminin and SDF‐1α can recruit endogenous stem cells and facilitate their differentiation into blood vessels by providing a supportive microenvironment for cell attachment and proliferation.

To further confirm that these regenerative processes occurred in a temporally coordinated manner, sequential immunostaining was conducted at different time points (1 d, 3 d, 7 d) in both the control and L100 S200 hydrogel groups (Figure S19, Supporting information). CXCR4 and CD90 staining revealed early recruitment of stem cells at 1 d, which further increased by 3 d. Subsequently, laminin and CD31 expression intensified from 3 d to 7 d, indicating enhanced ECM deposition, vascular maturation, and host integration. These results confirm that the PPLS hydrogel system promotes a stepwise regenerative sequence—from stem cell recruitment and adhesion to vascular differentiation—mimicking the intrinsic healing cascade.

### 2.6 Angiogenic Effects of the PPLS Hydrogel in a Hindlimb Ischemia Model

**Figure 7:**
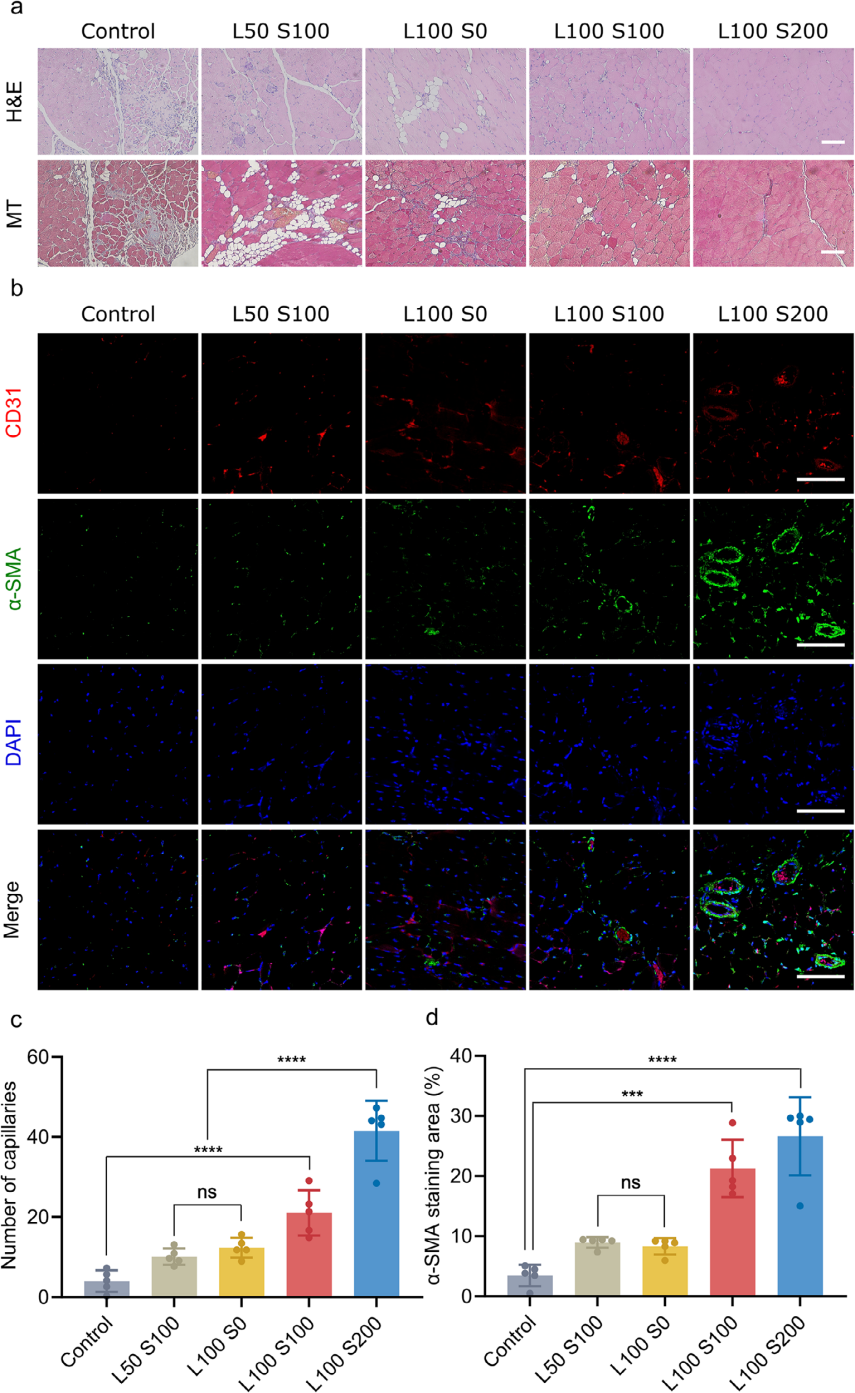
In vivo effects of regeneration on hind limb ischemia through angiogenesis. a) H&E and Masson's trichrome staining images 21 d post‐operation. Scale bar = 100 µm. b) Immunofluorescence images 21 d post‐operation. Scale bar = 200 µm. c,d) Quantification of the number of capillaries and the percentage of the α‐SMA‐stained area (n = 5). **p < 0.001, ***p < 0.0005, and ****p < 0.0001.

As previously noted, the L100 S200 group exhibited higher levels of angiogenesis than the other groups did; thus, tissue staining in this group was expected to reveal significantly reduced fibrosis and elevated expression of angiogenic markers. The mice were euthanized, and the hind‐limb muscles were harvested after 21 d to assess the angiogenic effects of the PPLS hydrogel. Insufficient tissue regeneration can lead to prolonged inflammation, necrosis, and, ultimately, tissue fibrosis.^[^ ^50^, ^51^ ^]^ Therefore, inflammatory cell infiltration and the morphological state of the ischemic muscle were analyzed using Hematoxylin and Eosin (H&E) staining (**Figure** **7**a; Figure S20, Supporting information). Compared with the other groups, the control and L50 S100 groups showed a notable presence of inflammatory cells within the muscle tissue, whereas the L100 S200 group had fewer cells. Similarly, severe muscle degeneration was evident in the ischemic muscles of the control and L50 S100 groups; however, L100 S200 mitigated muscle changes more effectively than the other groups did. We assessed the extent of fibrosis in the ischemic hind‐limb using Masson's trichrome (MT) staining (Figure 7a; Figure S20, Supporting information). A substantial amount of collagen was deposited in the control group, with a gradual decrease in intensity observed from L50 S100, L100 S0 and L100 S100, to L100 S200, with the latter exhibiting even lower levels. Blood vessel formation was also assessed on the basis of the expression levels of the angiogenesis markers CD31 and α‐SMA (Figure 7b–d). Compared with the other groups, the L100 S200 group showed a higher intensity than the other groups, suggesting more frequent angiogenesis. The L100 S200 group exhibited the most pronounced regenerative outcomes among all formulations, showing reduced inflammation and fibrosis together with robust vascular regeneration. This improvement likely resulted from the synergistic action between SDF‐1α‐mediated stem cell recruitment and laminin‐supported cell adhesion and differentiation, which collectively promoted efficient tissue repair in ischemic muscle.

## 3 Conclusion

A new hydrogel platform was developed to mimic intrinsic regeneration, characterized by stem cell homing, adhesion, proliferation, and differentiation; it was specifically designed to recruit and train stem cells within the hydrogel as a promising strategy for tissue regeneration. This hydrogel system was prepared by simple mixing of laminin and SDF‐1α through different incorporation forces, where SDF‐1α induced stem cell homing and laminin promotes cell adhesion and proliferation. Upon single injection at the local site, the hydrogel underwent a sol‒gel transition, enabling the sustained release of SDF‐1α to recruit stem cells, along with the prolonged incorporation of laminin to support their retention and establish a regenerative microenvironment, thereby facilitating tissue regeneration. Laminin and SDF‐1α effectively interacted with polymers, enhancing their stability and improving stem cell recruitment, adhesion, and proliferation both in vitro and in vivo. In a hindlimb ischemia model, a single injection of hydrogel at the surgical site accelerated angiogenesis by optimizing laminin and SDF‐1α concentrations, resulting in stem cell infiltration and blood vessel formation. Overall, this injectable hydrogel encapsulates dual factors that mimic natural regeneration mechanisms, promoting stem cell homing and proliferation within the hydrogel over a long period. Collectively, the results demonstrate that the hydrogel modulates key cellular processes, contributing to improved repair outcomes in the ischemic model.

## 4 Experimental Section

### Materials

Hexachlorocyclotriphosphazene (Sigma‐Aldrich, MO, USA) was purified via sublimation at 60 °C under a vacuum. Poly(dichlorophosphazene) was synthesized following established procedures outlined in a previous study.^[^ ^52^ ^]^ Methoxy poly(ethylene glycol) with a molecular weight of 750 Da was used to replace α‐amino‐ω‐methoxy poly(ethylene glycol) (AMPEG) within the P backbone. L‐Isoleucine ethyl ester hydrochloride (IleOEt·HCl) (A&Z Food Additives, China) was dried at 60 °C under vacuum. Glutaric anhydride, polyethyleneimine with a molecular weight of 423 (423 PEI), 2‐aminoethanol (AEtOH), and isobutyl chloroformate (Sigma‐Aldrich, USA) were procured before the study.^[^ ^27^ ^]^ Tetrahydrofuran (THF) and trimethylamine (TEA) were dried through reflux over sodium metal and barium oxide, respectively, under dry nitrogen. All the other reagents were prepared from commercial suppliers and used as received.

### Synthesis of P

IleOEt·HCl (24.65 g, 125.98 mmol) was dissolved in anhydrous THF. The mixture was stirred in a dry ice bath, and 50 mL of dried TEA was added. Poly(dichlorophosphazene) (10 g, 86.29 mmol) was dissolved in dry THF and introduced into the reactor. The reaction temperature was maintained at 50 °C for 24 h. The polymer underwent further reaction with the addition of ethanolamine (61.08 g, 17.26 mmol) and AMPEG (36.88 g, 49.19 mmol) for an additional 48 h. The resulting polymer solution was filtered, and the solvent was removed using a rotary evaporator. The polymer was precipitated by pouring it twice into n‐hexane (Daejung, Korea), followed by purification using a dialysis membrane (MWCO: 10–12 kDa; Spectrum Life Sciences, USA) against methanol and distilled water for 3 d each. The refined polymer solution was then filtered through a 0.45 µm syringe filter and freeze‐dried to produce aminoethanol‐linked poly(organophosphazene) (P‐OH). Anhydrous THF was added to P‐OH (18.93 g, 20.96 mmol) to facilitate the conjugation of the carboxylic acid group. Glutaric anhydride (4.78 g, 41.92 mmol) and N,N‐dimethylaminopyridine (5.13 g, 41.92 mmol) were dissolved in dry THF and added to the P‐OH solution. The mixture was then stirred and incubated at 45 °C for 24 h. Subsequently, the polymer was dialyzed against methanol and distilled water, for 3 d each; a dialysis membrane was used to remove impurities before freeze‐drying.

### Synthesis of PP

P (7 g, 3.27 mmol) was dissolved in chloroform (CHCl3), and the solution was cooled to 0 °C. Isobutyl chloroformate (0.447 g, 3.27 mmol) and TEA (0.663 ml, 6.55 mmol) were slowly added to the solution and stirred for 30 min to activate the carboxyl groups. The resulting mixture was then added to 423 PEI (9.694 g, 22.92 mmol) dissolved in chloroform and stirred for 24 h at 0 °C, followed by additional stirring for 48 h at 40–50 °C. The reaction mixture was concentrated and precipitated using a 1 M KF solution. Finally, the obtained products were purified using a dialysis membrane (MWCO: 12–14 kDa) against methanol and deionized water for 4 d each before lyophilization to yield PP. The structure of PP was determined via ^1^H‐NMR and ^13^C‐NMR analyses (Bruker Advance 400 MHz Fourier transform mode), with the polymer dissolved in deuterated chloroform. ^1^H NMR (CDCl_3_, δ, ppm): 0.92–1.06 (6H, –CH_3_ and –CH_2_CH_3_ of IleOEt), 1.07–1.40 (3H, –OCH_2_CH_3_ of IleOEt), 1.40–1.62 (2H, –CH_2_CH_3_ of IleOEt), 1.62–1.85 (1H, –CH(CH_3_)CH_2_CH_3_ of IleOEt), 2.25–2.65 (4H, –CH_2_CH_2_– of glutaric acid), 2.70–3.10 (broad, –NH–CH_2_–CH_2_– of PEI), 2.80–3.20 (4H, –NHCH_2_CH_2_O– of AMPEG), 3.38 (3H, –OCH_3_ of AMPEG), 3.50–3.92 (≈62H, –(CH_2_CH_2_O)_11_– of AMPEG), 3.92–4.05 (1H, –NH–CH– of IleOEt), 4.11–4.40 (4H, –OCH_2_CH_3_ of IleOEt). ^13^C‐NMR (CDCl_3_, δ, ppm): a characteristic signal at 30.94 ppm corresponding to the α‐methylene carbon adjacent to the free –COOH of P was used to monitor conjugation. This peak disappeared after PEI conjugation, indicating the formation of amide linkages between the glutaric anhydride and PEI. The amine groups in the PP were quantified using a ninhydrin assay kit (Pierce, Rockford, IL, USA).

### Preparation of the PP Solution

PP was dissolved in Dulbecco's phosphate‐buffered saline (DPBS; Welgene, Seoul, Korea) to produce a 10 wt.% solution.

### Preparation of the PPL or PPS Solution

Initially, PP was dissolved in DPBS to make a 12 wt.% solution. Then, 100 µg mL^−1^ laminin (Invitrogen, MA, USA) or 200 ng mL^−1^ SDF‐1α (R&D Systems, USA) was added to the mixture, and the mixture was incubated at 4 °C for 30 min. The final PP concentration was adjusted to 10 wt.%.

### Preparation of the PPLS Solution

Laminin (100 µg mL^−1^) and SDF‐1α (200 ng mL^−1^) were combined and incubated at 4 °C for 30 min. The mixture was added to a 12 wt.% PP solution and incubated at 4 °C for an additional 30 min, resulting in a final PP concentration of 10 wt.%.

### Rheological Analysis

Rheological measurements of PP, PPL, and PPLS were performed using a rheometer (MSC 102, Anton Paar, Graz, Austria). Hydrogel solutions were prepared at a concentration of 10 wt.%. Oscillatory frequency sweeps (1 Hz, 10% strain) over a temperature range of 4–60 °C were used to determine the sol–gel transition. To evaluate shear‐thinning properties, hydrogels were subjected to shear stresses from 0.1 to 100 Pa at 1 Hz at 25 °C and 37 °C. Self‐healing ability was examined by cyclically applying low strain (1%) and high strain (150%) at 1 Hz. The corresponding viscoelastic moduli were obtained from the rheometer's analysis software.

### FT‐IR

FT‐IR spectra were recorded on a Frontier FT‐IR spectrometer (PerkinElmer, USA) equipped with an ATR MIRacle Diamond accessory (PIKE Technologies). Each spectrum was collected in the range of 4000–500 cm^−1^ with a resolution of 4 cm^−1^ by averaging 32 scans.

### GPC

GPC analysis was performed on a Waters Alliance e2695 system (Waters, USA) equipped with a refractive index detector using three Waters Styragel columns (HR3, HR4, and HR5E) connected in series. THF (B&J) was used as the mobile phase at a flow rate of 1.0 mL min^−1^ and a column oven temperature of 35 °C. The system was calibrated with narrow polystyrene standards ranging from 1060 to 1 320 000 g mol^−1^. Polymer samples were dissolved in THF (2 mg mL^−1^) and filtered through a 0.45 µm PTFE syringe filter prior to injection.

### In Vitro Cell Viability Test

NIH3T3 cells (Korean Cell Line Bank, Korea) and mouse mesenchymal stem cells (mMSCs; Cyagen Biosciences Inc., USA) were seeded in 96‐well cell culture plates (SPL Life Sciences, Korea) at a density of 1 × 10^4^ cells/well and incubated for 24 h in Dulbecco's modified Eagle's medium (DMEM; Welgene, and Gibco, USA) supplemented with 10% fetal bovine serum (FBS; Welgene) and 1% penicillin/streptomycin (Gibco, USA). The culture medium was subsequently replaced with serum‐free media containing varying concentrations of PP (0 to 1 mg mL^−1^). After 24 h of incubation, 100 µg of thiazolyl blue tetrazolium bromide (MTT; 98%, Alfa Aesar, MA, USA) dissolved in media was added to each well, followed by incubation for an additional 3 h. The supernatants were then removed, and 100 µL of dimethylsulfoxide (DMSO; Daejung) was added to dissolve the formazan crystals. The absorbance of the resulting solution was measured at 570 nm using a SpectraMax 340 Microplate Reader (Molecular Devices, CA, USA).

### Measurement of Sizes and Surface Charges

The sizes and zeta potentials of laminin, PP, LS, and PPLS were determined using a Zetasizer Nano ZS (Malvern Instruments Ltd., Worcestershire, UK) at 25 °C. The polymer was dissolved at 1 wt.% in DPBS (Welgene), and measurements were conducted five times for each sample.

### TEM

The morphology of the complexes was analyzed using an FEI Tecnai F20 G^2^ TEM (operated at 200 kV) equipped with a Gatan digital camera. Copper grids (200‐mesh) were glow‐discharged with Ar/O_2_ plasma for 30 s before sample loading. A 10 µL of each sample solution was applied to the prepared grids and allowed to adsorb for 1 min. The grids were then negatively stained with UranyLess (Electron Microscopy Sciences, USA) for 1 min, followed by three washes with distilled water (30 s each) and completely air‐dried at room temperature prior to imaging.

### In Vitro Laminin and SDF‐1α Release Test

200 µL of the PPLS solution was transferred to a Millicell (12 mm; Millipore, USA) and heated at 37 °C for 30 min to induce hydrogel formation. Subsequently, 4 mL of DPBS was slowly added, and the mixture was incubated in a water bath (KMC‐12055W1, Vision, Korea) at 37 °C with shaking at 50 rpm. DPBS was periodically replaced with fresh buffer, and the released amount of laminin and SDF‐1α was quantified using a Micro BCA protein assay kit (Thermo Fisher Scientific, Korea) and a Mouse CXCL12/SDF‐1 alpha Quantikine ELISA kit (R&D Systems).

### Measurement of Hydrogel Structure with Cryo‐SEM

mMSCs were cultured in DMEM (Gibco, USA) supplemented with 10% FBS and 1% penicillin–streptomycin at 37 °C in a 5% CO_2_ incubator. Cryo‐SEM experiments were performed using a Quanta 3D FEG microscope (FEI, Netherlands) equipped with an Alto 2500 cryo‐transfer system (Gatan, UK). PPS and PPLS hydrogels were mixed with 1 × 10⁶ cells per sample at a final concentration of 10 wt%. For comparison, PP, PPL, PPS, and PPLS hydrogels were prepared at the same final concentration (10 wt%) without cell incorporation. The prepared samples were quickly frozen in liquid nitrogen in a pre‐evacuated freezing chamber (10^−5^ mbar, –190 °C). The hydrogel samples were fractured with a cold blade and etched to sublimate surface ice. Metal deposition was performed by sputtering with a 3 mA current, and the samples were transferred to a microscope chamber preset to 10^−5^ mbar at –190 °C. Cryo‐SEM images were acquired at 5 keV and 11.8 pA.

### In Vitro Cell Attachment Test

mMSCs (1 × 10^6^) were mixed with PPS and PPLS hydrogels and incubated at 37 °C for 30 min. DMEM supplemented with 10% FBS and 1% penicillin/streptomycin was added to the hydrogel group, followed by an incubation period of 3 d. Cell viability and attachment were assessed using calcein‐AM and ethidium homodimer‐1 staining (Invitrogen), and the cells were observed under a microscope (Zeiss, Germany). Data analysis was conducted using ImageJ software.

### In Vitro Cell Proliferation Assay (MTT Assay)

mMSCs were seeded in 96‐well plates at a density of 1 × 10^4^ cells per well and incubated for 24 h in DMEM supplemented with 10% FBS and 1% penicillin‒streptomycin. The culture medium was then replaced with serum‐free medium containing 10 mg mL^−1^ PPS or PPLS. After incubation for 1, 3, and 5 d, 100 µg of MTT dissolved in media was added to each well, followed by incubation for 3 h. The supernatants were then removed, and 100 µL of DMSO was added to dissolve the formazan crystals. The absorbance of the resulting solution was measured at 570 nm by using a SpectraMax 340 Microplate Reader (Molecular Devices).

### In Vitro Cell Proliferation Assay (Live/Dead Assay)

mMSCs (5 × 10^5^) were combined with the PPS or PPLS hydrogels and incubated at 37 °C for 30 min. Subsequently, DMEM supplemented with 10% FBS and 1% penicillin/streptomycin was added to the hydrogel groups and the samples were incubated for an additional 1, 3, 5, or 7 d. Cell proliferation was evaluated using calcein‐AM and ethidium homodimer‐1 staining (Invitrogen), and observed under a microscope (Zeiss). Data analysis was conducted using ImageJ software.

### Transwell Migration Assay

mMSC migration was conducted using a Transwell assay that consisted of a cell insert (8 µm; SPL Life Sciences). The 10 wt.% PP, PPS, PPL, and PPLS hydrogels were placed in the lower chamber with complete medium and mMSCs (1× 10^6^ per well) were seeded in the upper chamber with serum‐free medium. For CXCR4 inhibition, mMSCs were seeded onto the inserts in serum‐free medium supplemented with 5 µm of AMD3100 (Sigma Aldrich) and incubated for 30 min at 37 °C prior to the migration assay. After 48 h of incubation, the cells were stained with 0.2% crystal violet (Sigma Aldrich) and observed under a microscope (Nikon, Japan). The cells were counted using the ImageJ software.

### Scratch Assay

HUVECs (CEFO Co., Ltd, Korea) were seeded in a 24‐well plate (SPL Life Sciences) at a density of 1 × 10^5^ cells/well and cultured in RPMI‐1640 medium (HyClone Laboratories, UT, USA) supplemented with 10% FBS and 1% penicillin‒streptomycin at 37 °C in a 5% CO_2_ incubator. After culturing for 24 h to ≈80% confluence, each well was scraped using a scar scratcher (SPL Life Sciences) and washed once with DPBS. The hydrogel groups were treated with complete medium and incubated for 24 h. The migration rate was measured using ImageJ software, and the initial and final scratch areas were compared.

### Modeling of Hindlimb Ischemia

All mouse experimental protocols were approved by the Institutional Animal Care and Use Committee of the Korea Institute of Science and Technology. Six‐week‐old male BALB/c mice (Orient Bio, Seoul, Korea) were anesthetized with 3% isoflurane in a mixture of oxygen and nitrogen. Ligation of the proximal branches of the external iliac artery and vein, as well as the distal bifurcation into the saphenous and popliteal arteries and veins, was performed on the right limbs using 5–0 silk sutures (AILEE, Korea). The midpoint of the ligated area was dissected. Following surgery, 50 µL of hydrogel was immediately injected into the ischemic region of the hindlimb using a 31G insulin syringe.

### Laser Doppler Imaging

Angiogenesis was analyzed via a laser Doppler perfusion imager (LDPI; Moor Instruments, UK) at 0, 7, 14, and 21 d postoperatively. A heating pad was used to maintain the body temperature of the mice during LDPI scanning. Blood perfusion from the knee joint to the toe was quantified by analyzing the color images; specifically, the perfusion ratio was calculated by dividing the mean value of the left ischemic hindlimb by that of the right normal hindlimb.

### In Vivo Stem Cell Distribution

Hindlimb tissues were extracted after 21 d and fixed with 4% paraformaldehyde for 24 h. The tissues were embedded in paraffin and cut into 5‐µm‐thick sections. The sections were deparaffinized, rehydrated, and washed twice with distilled water. Antigen retrieval was performed using a retrieval buffer (Dako, Denmark) for 10 min at 100 °C, followed by three washes with distilled water. The samples were permeabilized with phosphate‐buffered saline with Tween 20 (PBST) for 10 min and incubated with primary antibodies against CD90 (1:100; MA1‐81491; Invitrogen), CD34 (1:100; MA1‐22646; Invitrogen), or Ang‐1 (1:100; except using Alexa Fluor 555 secondary antibody) diluted in antibody diluent (Dako) for 24 h at 4 °C. After three washes with PBST, the sections were incubated with secondary antibodies conjugated to Alexa Fluor 488 (1:100; 4408S; Cell Signaling Technology, MA, USA) for CD90 and CD34, or Alexa Fluor 555 (1:100; 4413S; Cell Signaling Technology) for Ang‐1, for 1 h at room temperature. Finally, the tissues were counterstained with 4′,6‐diamidino‐2‐phenylindole (DAPI; Vector Laboratories, USA) and examined under a confocal laser scanning microscope (Zeiss).

### In Vivo Temporal Progression of Regeneration

For early‐stage analysis (1 d and 3 d), samples were stained with anti‐CD90 (1:100; 105 301; BioLegend) and anti‐CXCR4 (1:100; ab124824; Abcam) antibodies to evaluate mesenchymal stem cell recruitment and chemokine receptor expression. For later‐stage analysis (3 d and 7 d), sections were stained with anti‐laminin (1:100; L9393, Sigma) and anti‐CD31 (1:100; 10 251, BioLegend) antibodies to assess extracellular matrix deposition and vascular formation. Alexa Fluor 488 (1:100; A21206, Invitrogen) and Alexa Fluor 594 (1:100; A21209, Invitrogen) were used as secondary antibodies. Nuclei were counterstained with DAPI (Vector Laboratories). Fluorescence images were obtained using a confocal laser scanning microscope (Zeiss).

### In Vivo Laminin Holding Test

Green fluorescent laminin (Cytoskeleton, Inc., USA) was mixed with PP and SDF‐1α for visualization. A 50 µL volume of each hydrogel subsequently injected into the hindlimbs of the mice. The remaining laminin was monitored at predetermined time points using an IVIS Spectrum Imaging System (Caliper Life Sciences, USA).

### Histological and Immunohistological Analysis

Hindlimb tissues were washed with PBS and fixed with 4% paraformaldehyde for 24 h. The tissues were then embedded in paraffin and cut into 4 µm‐thick sections. The sections were deparaffinized, dehydrated, and stained with hematoxylin and eosin (H&E) and Masson's trichrome (MT). Images were obtained using an inverted microscope (Nikon, Tokyo, Japan).

The sectioned tissues were used for immunofluorescence staining; for this, they were deparaffinized, rehydrated, and washed twice with distilled water. The tissues were subsequently treated with antigen retrieval buffer (Dako) for 10 min at 100 °C and washed three times with distilled water. The samples were permeabilized with PBST (Biosesang, Korea) for 10 min and incubated with primary antibodies against CD31 (1:100; ab28364; Abcam, UK) and αSMA (1:100; ab7817; Abcam) diluted in an antibody diluent (Dako) for 2 h at room temperature. After washing three times with PBST, the samples were treated with secondary antibodies against Alexa Fluor 555 (1:100; 4413S; Cell Signaling Technology) and Alexa Fluor 488 (1:100; 4408S; Cell Signaling Technology) for 1 h at room temperature. The tissues were counterstained with DAPI (Vector Laboratories) and observed using a confocal laser scanning microscope (Zeiss).

### Statistical Analysis

All the results were analyzed using GraphPad  Prism software (GraphPad Software, Inc., La Jolla, CA, USA) and are presented as the means ± standard deviations. One‐way ANOVA with post‐hoc Tukey's multiple comparison test was used to evaluate the significance of differences among the groups, with significance levels indicated as follows: *p < 0.01, **p < 0.001, ***p < 0.0005, and ****p < 0.0001. The data in Figure 3 were analyzed using Student's *t*‐test, with significance levels indicated as follows: *p < 0.05, ***p < 0.0005, and ****p < 0.0001.

### Ethics Approval Statement

All experiments with live mice were carried out in compliance with relevant laws and institutional guidelines of the Institutional Animal Care and Use Committee (IACUC) in Korea Institute of Science and Technology (KIST), and IACUC approved the experiment (approval number: 2022‐112‐6).

## Conflict of Interest

The authors declare no conflict of interest.

## Supporting information



Supporting Information

Supporting Information

Supporting Information

## Data Availability

The data that support the findings of this study are available from the corresponding author upon reasonable request.
