# Supplementary material for: A Bioinstructive Injectable Hydrogel for Enhancing Intrinsic Regeneration through Cell Recruitment and Training
Source: Adv Sci (Weinh). 2025 Dec 23;13(10):e14549. doi: 10.1002/advs.202514549 (PMC12915135; doi:10.1002/advs.202514549)
Supplement: Supplementary file 1 — Supporting Information [file ADVS-13-e14549-s003.pdf]

Supporting Information

A Bioinstructive Injectable Hydrogel for Enhancing Intrinsic Regeneration through Cell Recruitment and Training

*Yurim Kim, and Young-Min Kim**

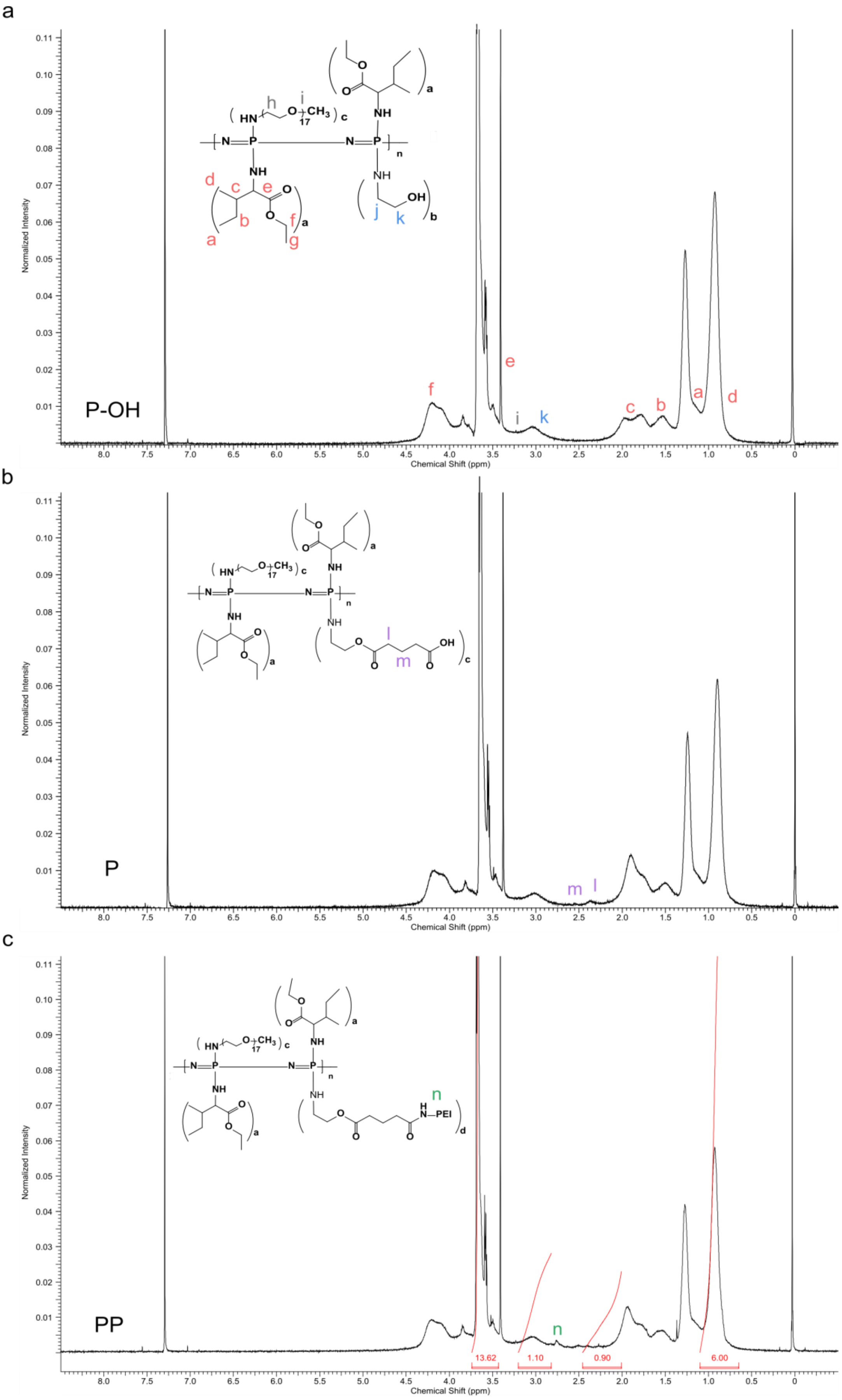

Figure S1. ^{1}H-NMR spectrum of a) P-OH, b) P, and c) PP.

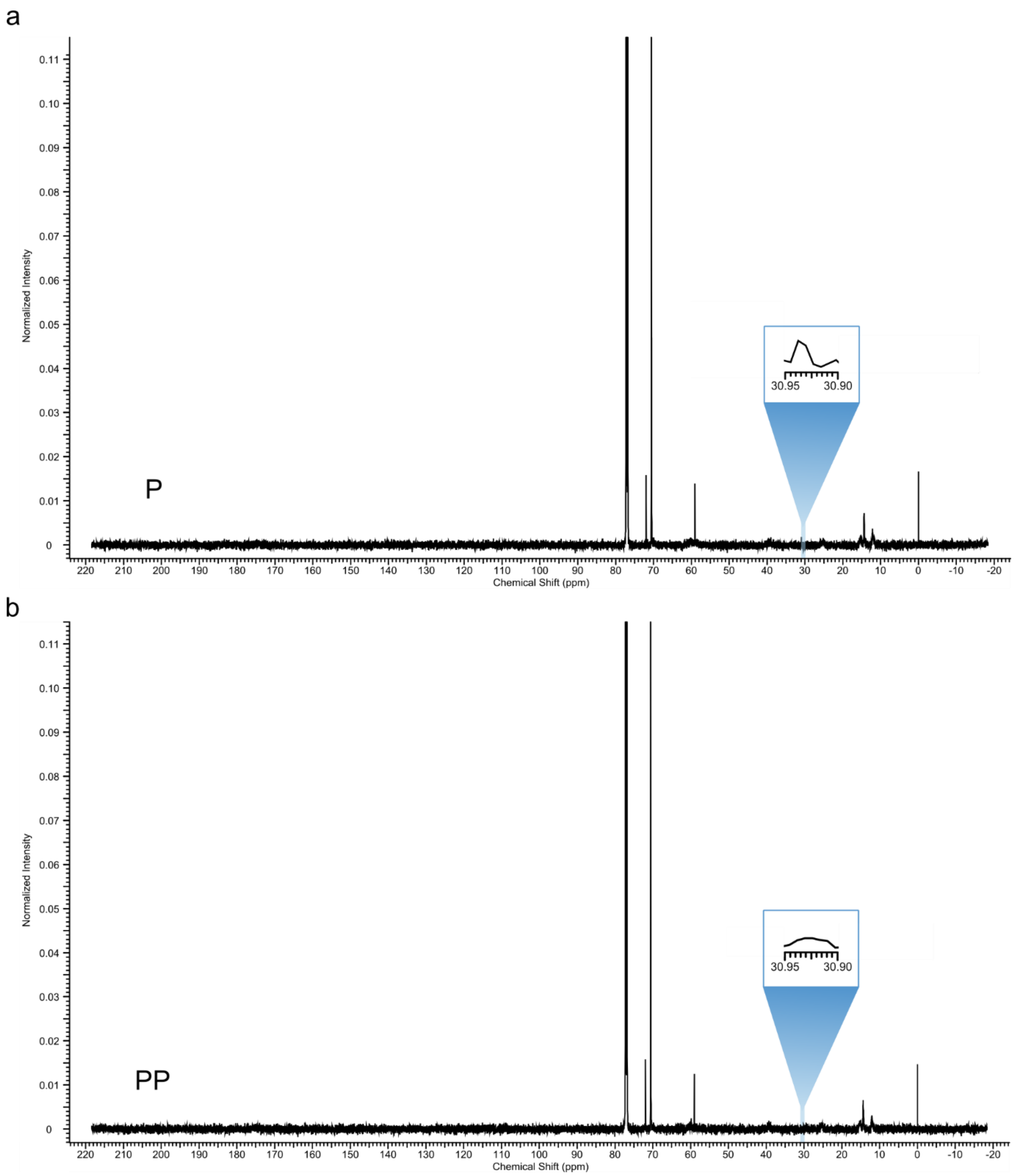

Figure S2. ^{13}C-NMR spectrum of a) P and b) PP.

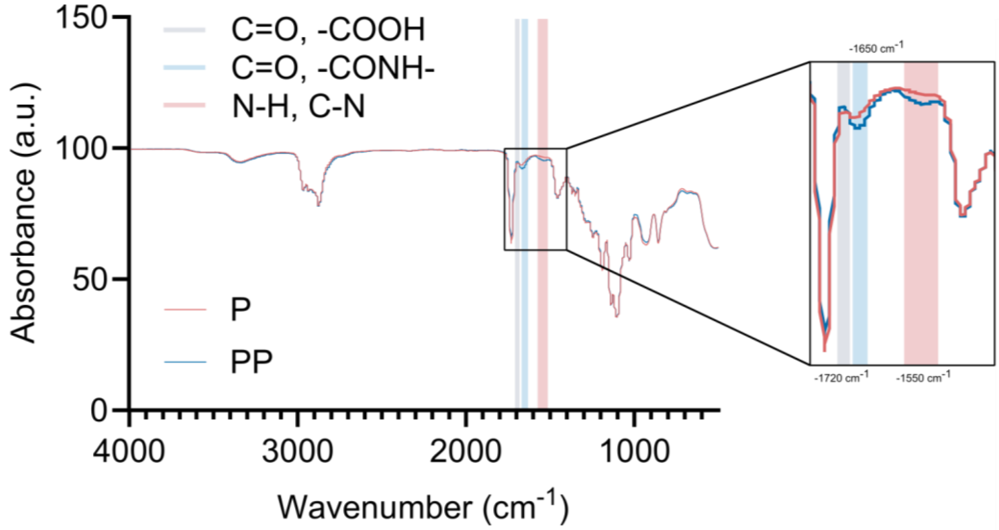

Figure S3. FT-IR spectra of P and PP hydrogels measured in the range of 4000-500 cm^{-1}.

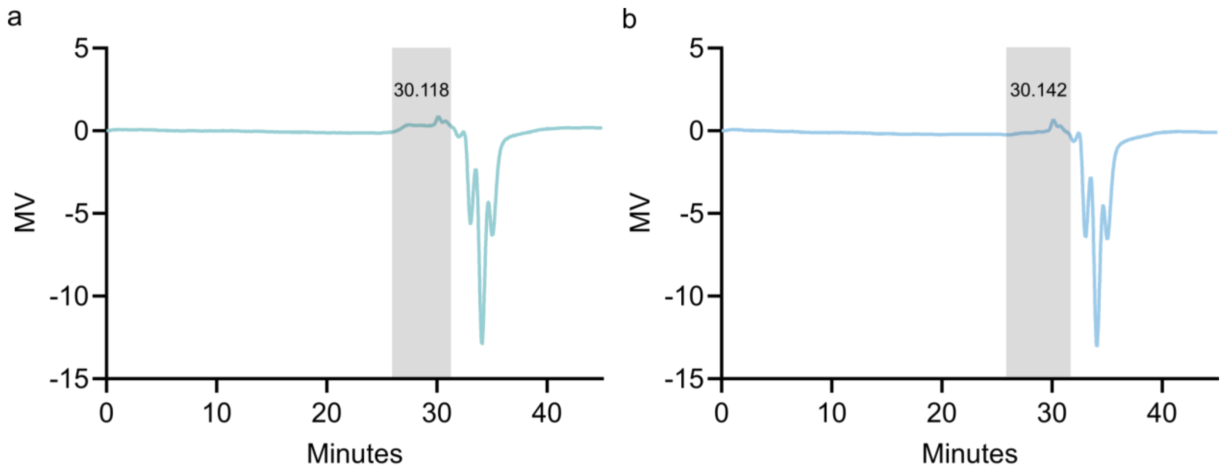

a

Mn	Mw	PDI
1867	2191	1.174

b

Mn	Mw	PDI
1289	1356	1.051

Figure S4. GPC chromatograms of a) P and b) PP in THF using polystyrene standards.

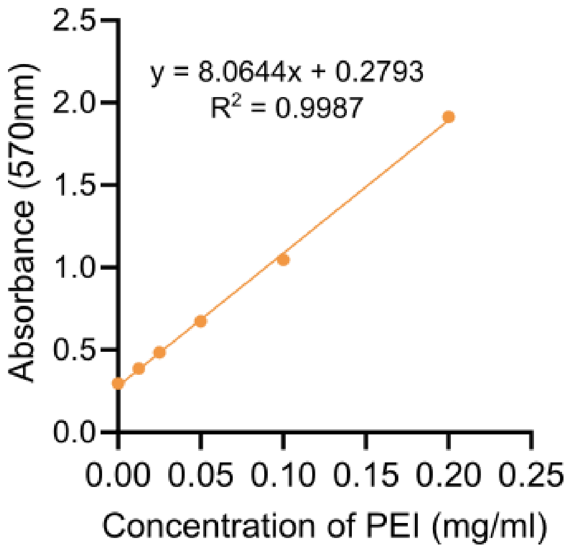

Figure S5. Standard curve of PEI obtained using a ninhydrin assay.

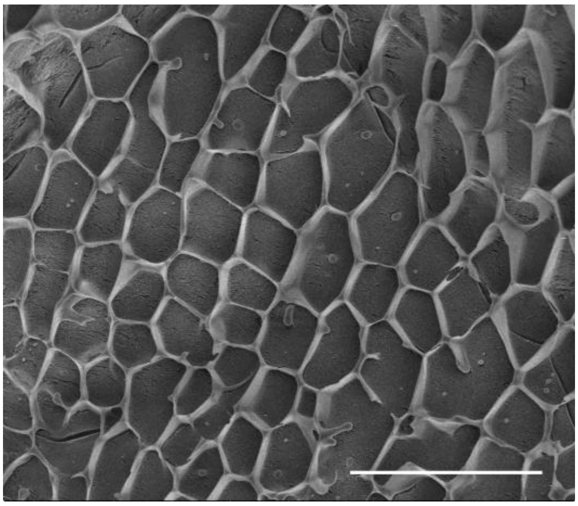

Figure S6. Cryo-SEM image of PP. Scale bar = 20 µm.

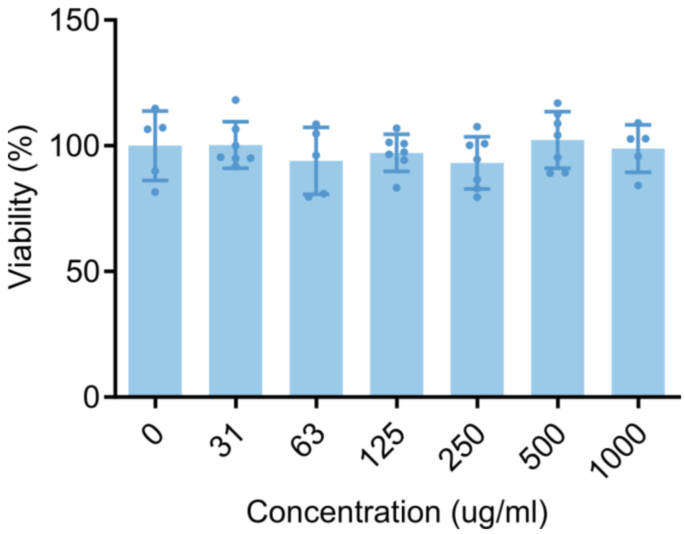

Figure S7. Cytotoxicity assessment of PP at various concentrations (0–1000 μg/mL) in NIH 3T3 cells.

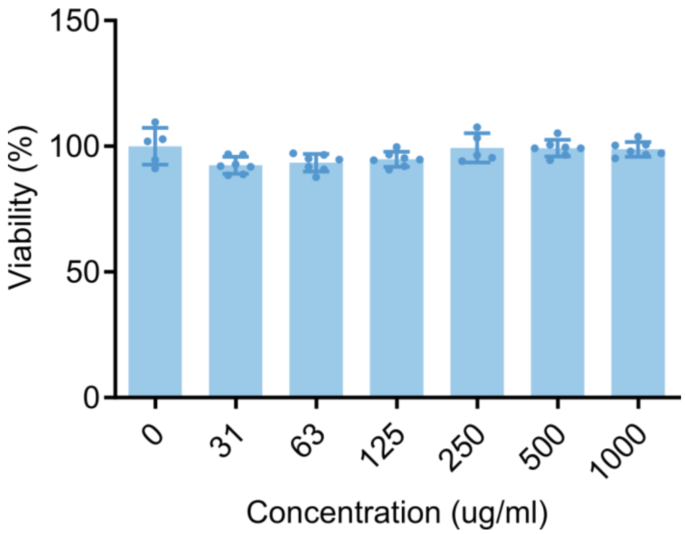

Figure S8. Cytotoxicity assessment of PP at various concentrations (0–1000 µg/mL) in MSCs.

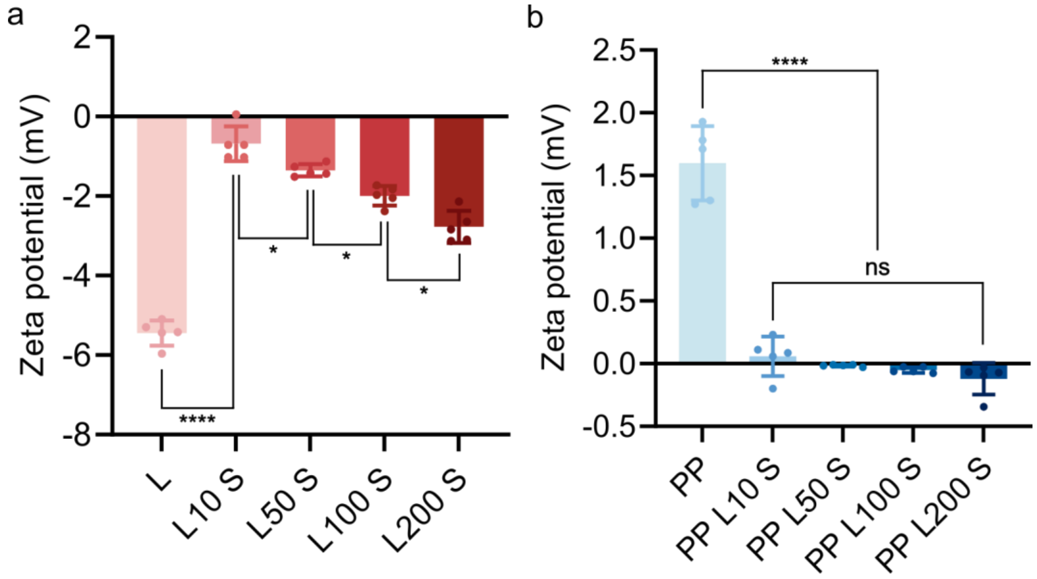

Figure S9. Zeta potential assessment of a) LS and b) PPLS at various concentration of laminin (10–200 µg/mL).

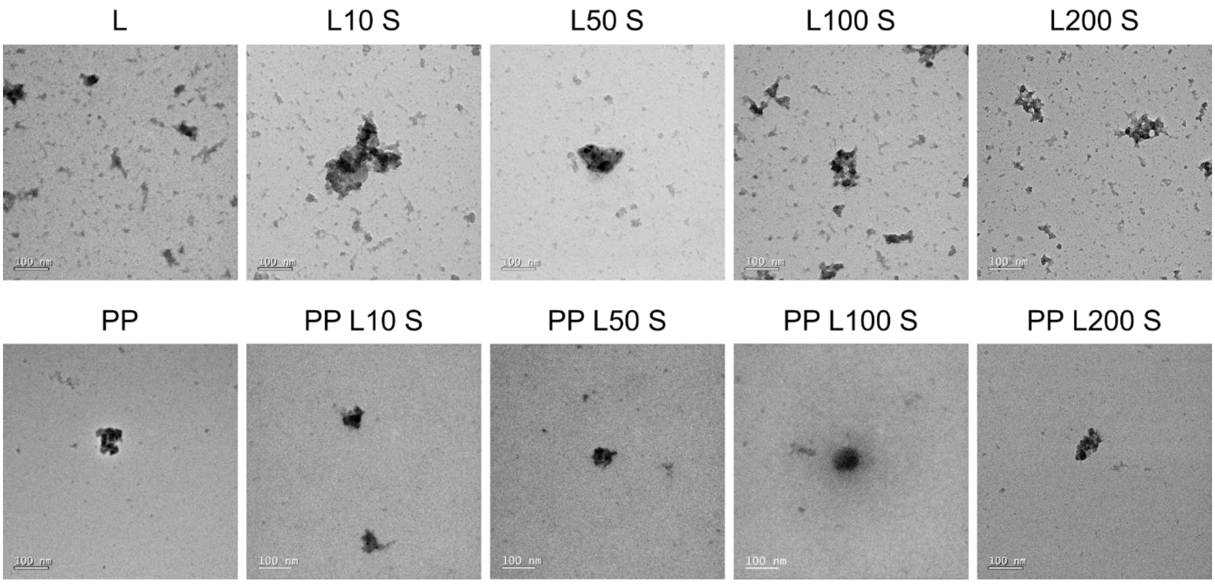

Figure S10. TEM image of LS and PPLS at various concentration of laminin (10–200 µg/mL). Scale bar = 100 µm.

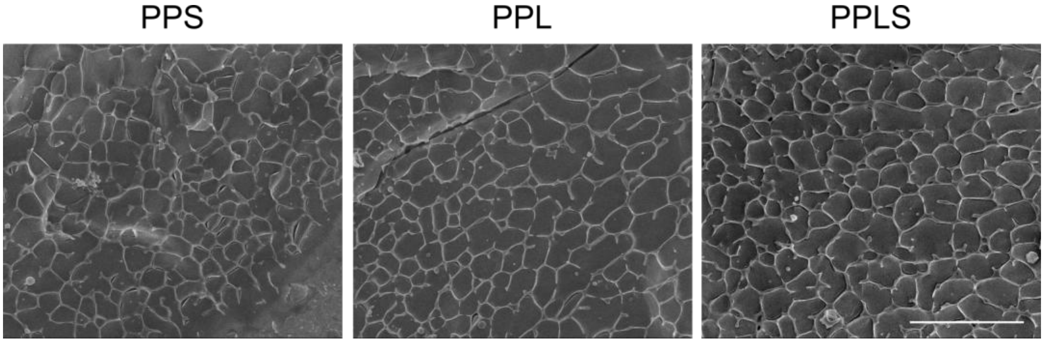

Figure S11. Cryo-SEM image of PPS, PPL, PPLS. Scale bar = 20 µm.

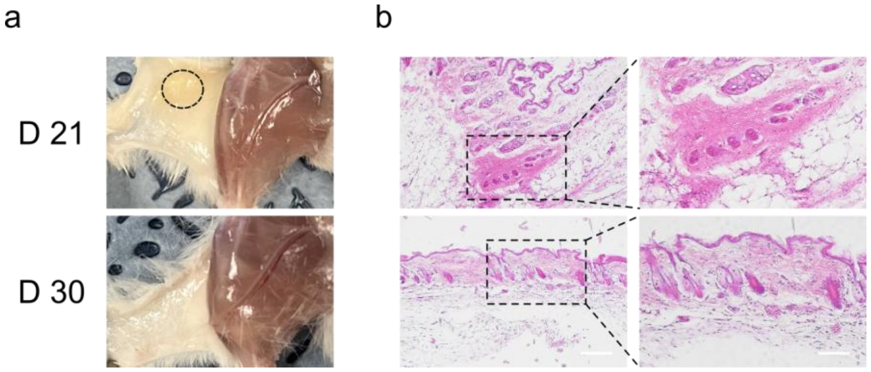

Figure S12. *In vivo* degradation and host integration of the PPLS hydrogel. a) *In vivo* image of the injection site 21 d and 31 d post-injection. (dotted circle: partial tissue-organized hydrogel). b) H&E staining images of the injection site 21 d and 31 d post-injection. Scale bar = 100 µm.

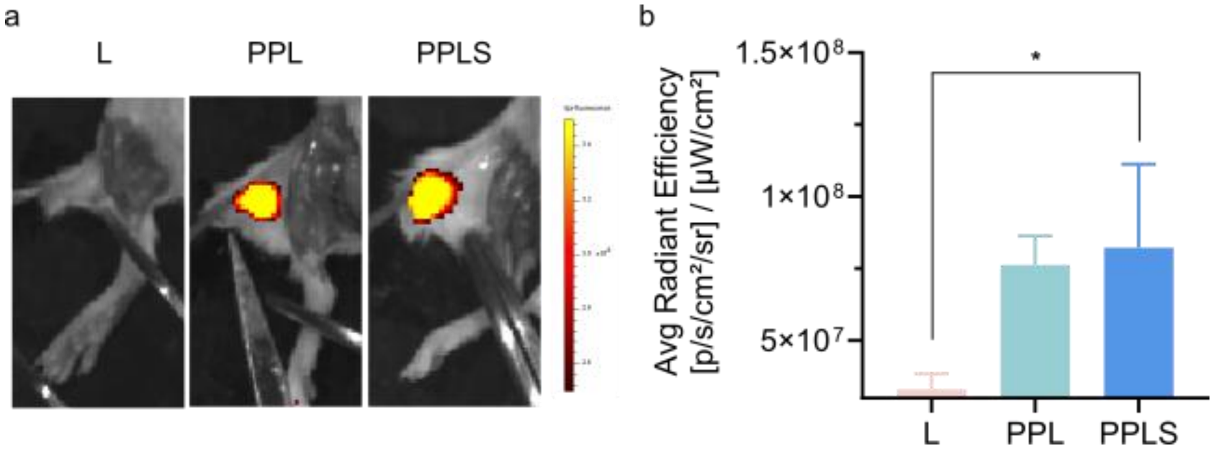

Figure S13. IVIS images of the remaining laminin 21d post-injection. *p < 0.05.

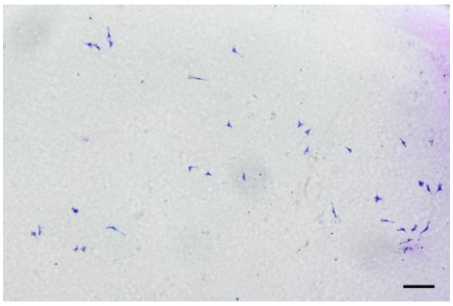

Figure S14. Schematic representation of the MSC migration assay through the PPLS hydrogel with the CXCR4 inhibitor AMD3100. Scale bar = 500 µm.

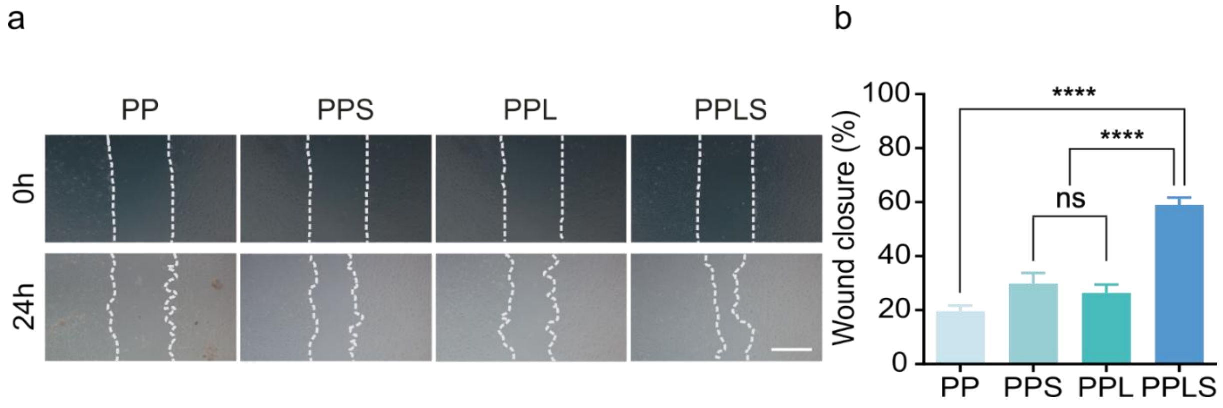

Figure S15. Effects of PP, PPS, PPL, and PPLS on HUVEC migration. Scale bar = 300 µm. ns = not significant, and ****$p < 0.0001$.

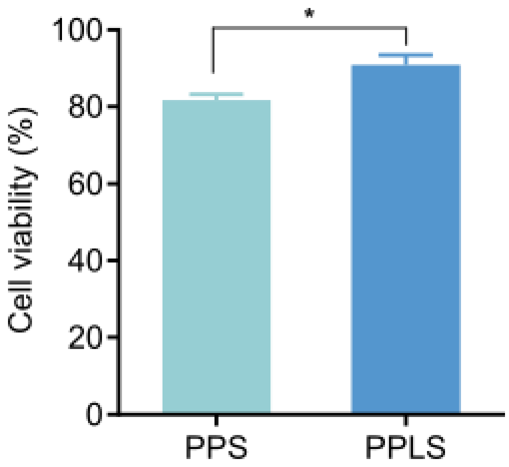

Figure S16. Quantitative analysis of live/dead staining images of PPS and PPLS. *$p < 0.05$.

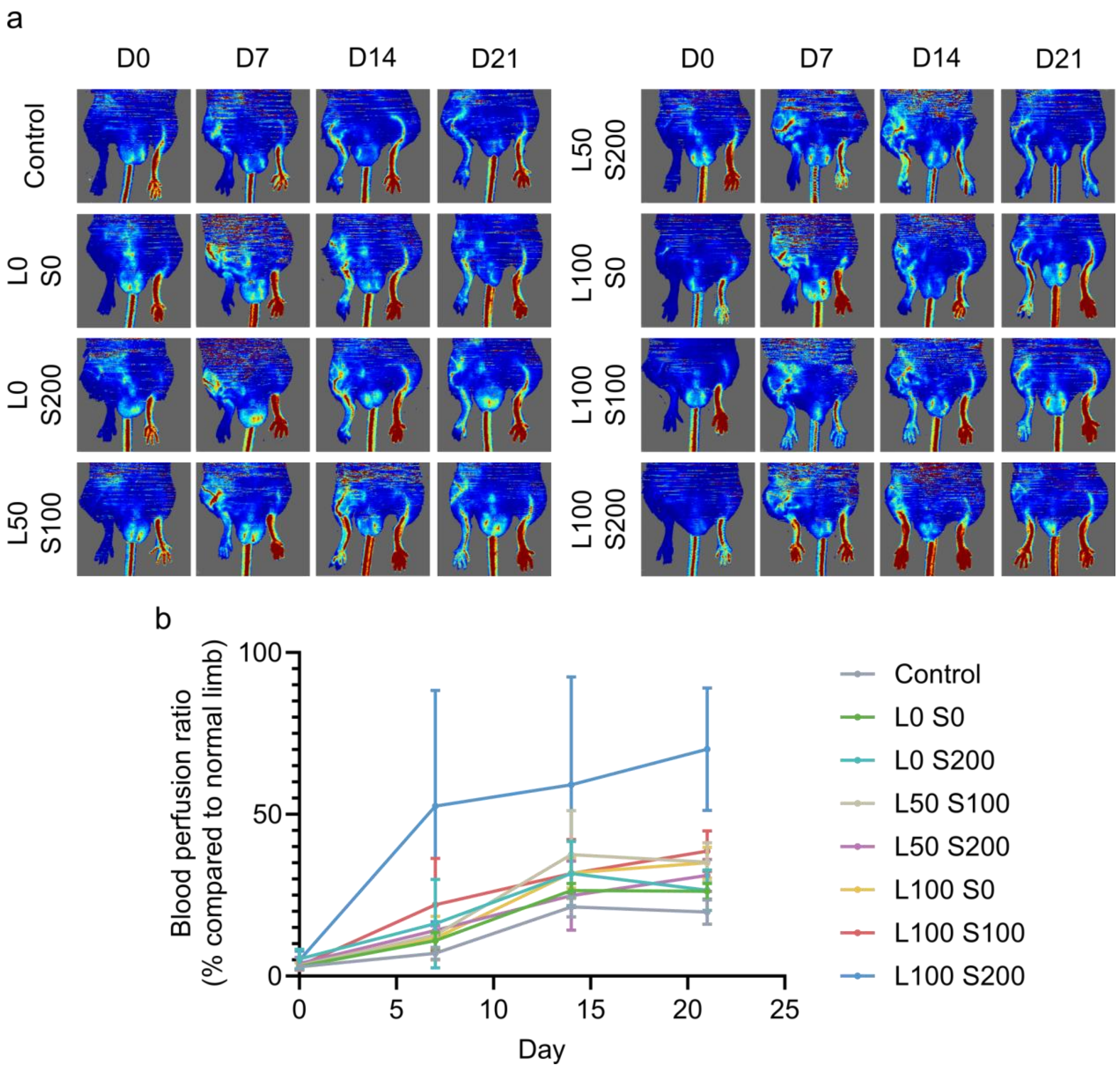

Figure S17. Evaluation of regenerative outcomes in a mouse hindlimb ischemia model with varying concentrations of laminin and SDF-1α. a) Laser Doppler perfusion imaging (LDPI) of the hindlimb ischemia model. b) Blood perfusion ratios at 0, 7, 14, and 21 d post-injection (n=3).

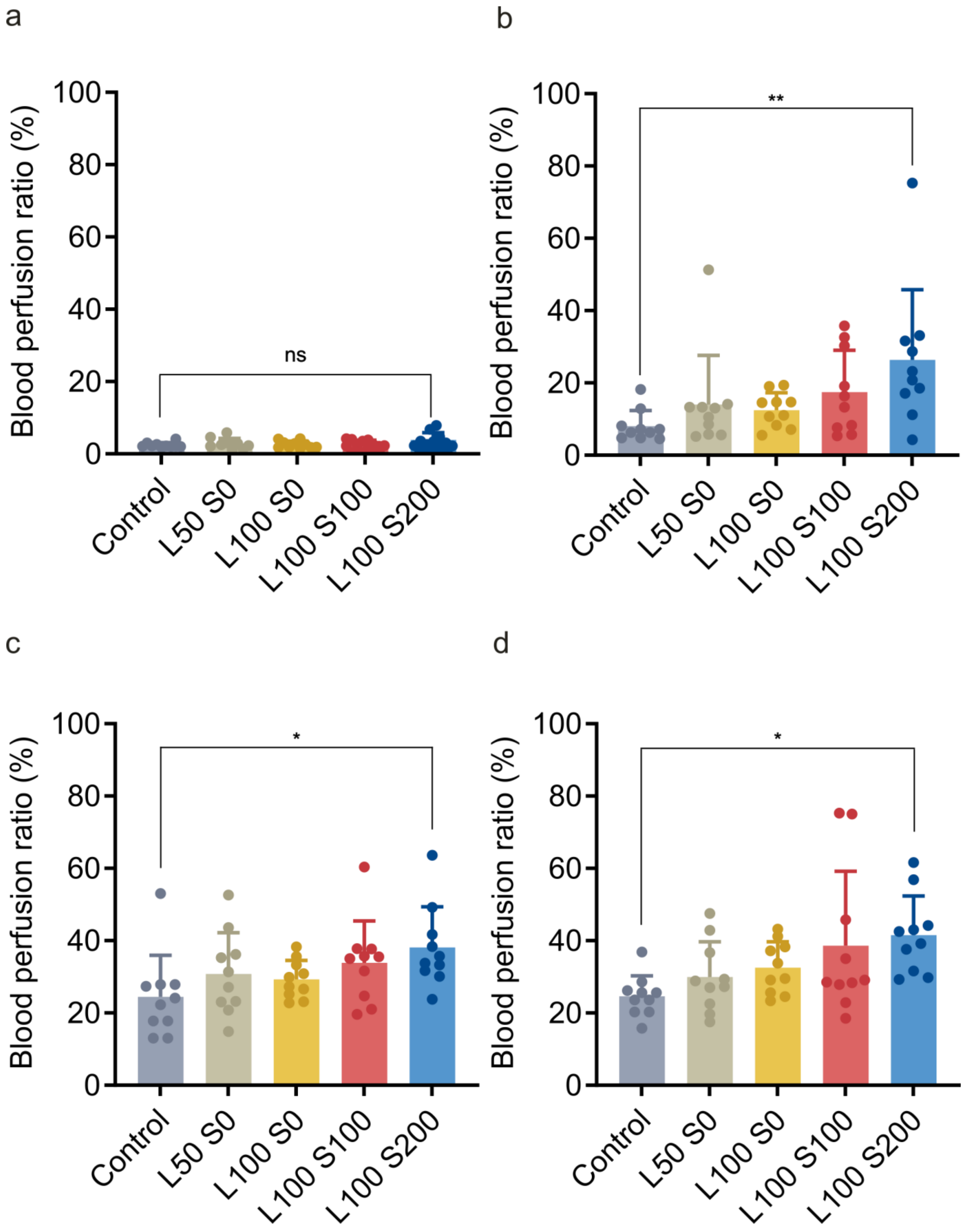

Figure S18. Quantitative analysis of the blood perfusion ratios on days a) 0, b) 7, c) 14, and d) 21. ns = not significant, *p < 0.01, and **p < 0.001.

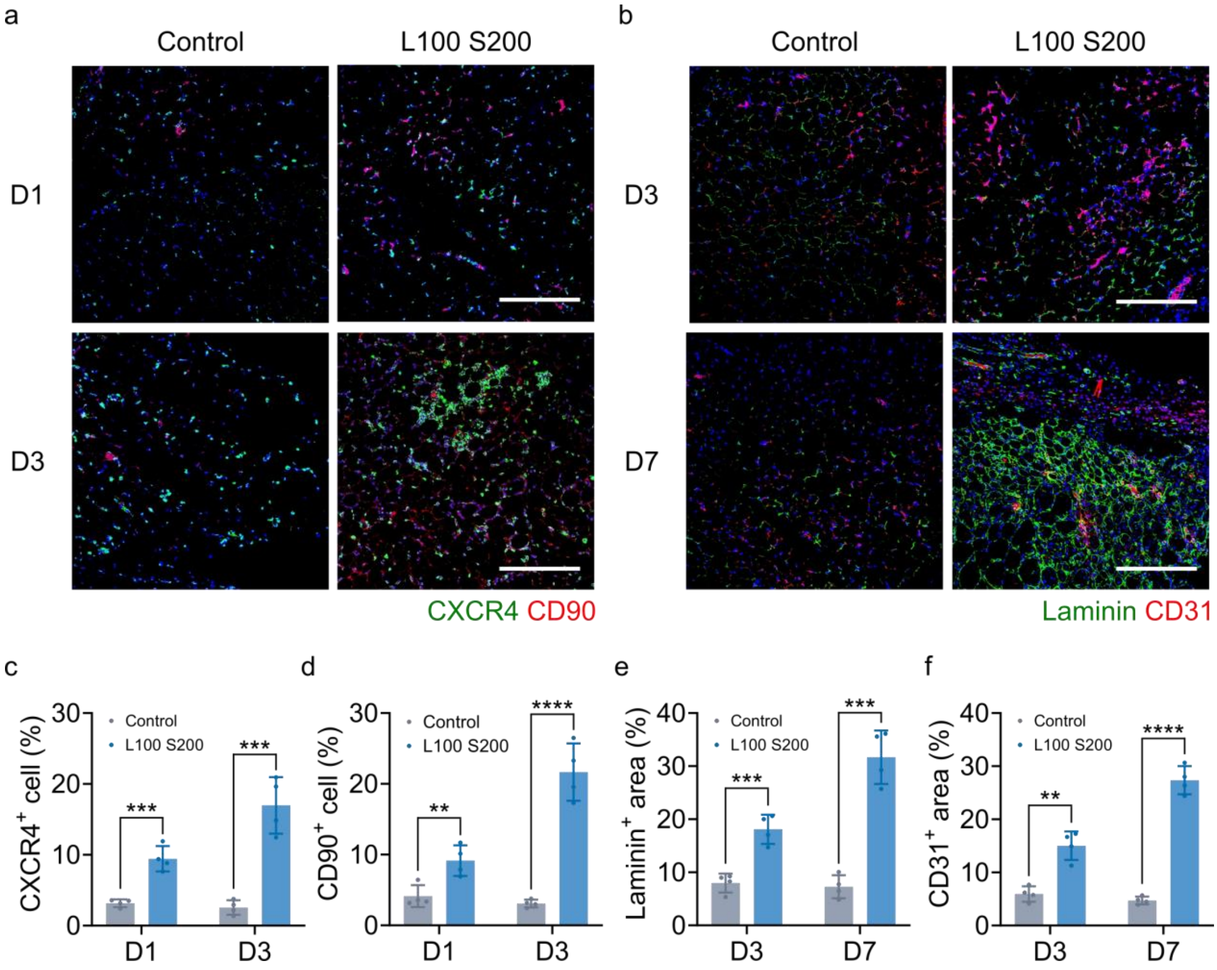

Figure S19. Sequential orchestration of cellular recruitment, ECM remodeling, and angiogenesis during early tissue regeneration. a-b) Immunofluorescence images of stem cell recruitment ($CXCR4^+$, $CD90^+$) and subsequent ECM and vascular remodeling ($Laminin^+$, $CD31^+$) at different time points post-injection. Scale bar = 200 μm. c-f) Quantification of the $CXCR4^+$ cells, $CD90^+$ cells, $Laminin^+$ area, and $CD31^+$ area (n=4). **$p < 0.001$, ***$p < 0.0005$, and ****$p < 0.0001$.

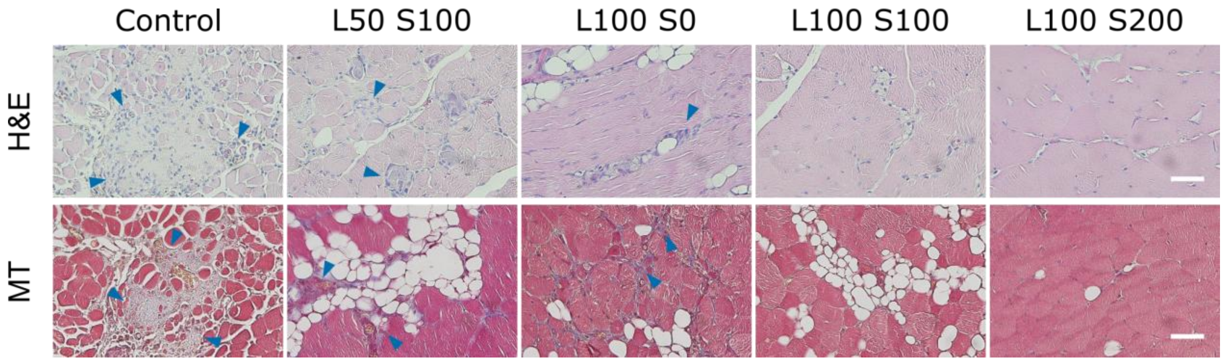

Figure S20. H&E and MT staining high magnification images. Blue arrowheads in H&E: inflammatory-cell infiltrates. Blue arrowheads in MT: collagen-rich fibrotic regions. Scale bar = 50 µm.

Movie S1. *In vitro* gelation behavior of PPLS hydrogel.

Injection of Nile Red–labeled PPLS solution into 37 °C water demonstrates the rapid sol–gel transition of the hydrogel upon exposure to physiological temperature.

Movie S2. *In vivo* gelation of PPLS hydrogel following subcutaneous injection.

Time-lapse recording showing the instant gel formation of PPLS after injection into the mouse hindlimb, confirming its thermosensitive and injectable characteristics.
